# From Tc^VII^ to Tc^I^; facile syntheses of bis-arene complexes [^99(m)^Tc(arene)_2_]^+^ from pertechnetate†
†Electronic supplementary information (ESI) available: Additional synthetic and characterization details (including HPLC, NMR, CV), and crystallographic details. CCDC 1017765, 1017766, 1017767, 1017768 and 1017769. For ESI and crystallographic data in CIF or other electronic format see DOI: 10.1039/c4sc02461c


**DOI:** 10.1039/c4sc02461c

**Published:** 2014-09-05

**Authors:** Michael Benz, Henrik Braband, Paul Schmutz, Jonathan Halter, Roger Alberto

**Affiliations:** a Department of Chemistry , University of Zürich , Winterthurerstr. 190 , CH-8057 Zürich , Switzerland . Email: ariel@chem.uzh.ch

## Abstract

Bis-arene complexes of technetium represent a fundamental class of organometallic compounds. Due to complex synthetic routes, no detailed insights into their properties have been reported so far. Reacting [^99^TcO_4_]^–^ with arenes in the exclusive presence of AlCl_3_ gives highly stable [^99^Tc(arene)_2_]^+^ in good yields. These complexes have extraordinarily high stabilities, where oxidation is found to occur at potentials higher than +1.3 V and reduction at potentials below –2 V *vs.* Fc/Fc^+^. The ^99m^Tc analogues are similarly synthesised by applying a novel ionic liquid extraction pathway. Complexes of ^99m^Tc with suitably functionalized arenes will represent new building blocks for bioorganometallic pharmaceuticals in molecular imaging.

## Abstract 2

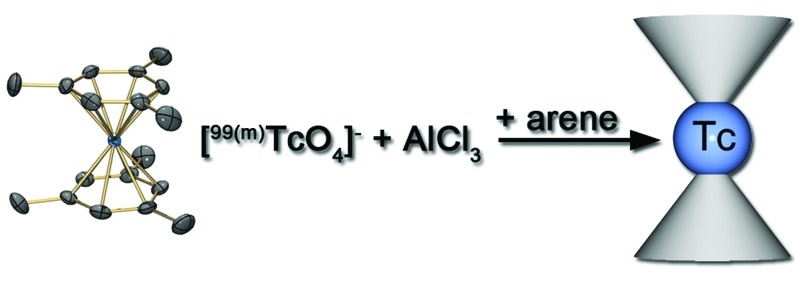
Bis-arene complexes of technetium represent a fundamental class of organometallic compounds. Detailed insights into their properties are obtained due to novel and convenient synthetic routes.

## Introduction

The realm of organometallic chemistry emerges from a relatively small number of basic ligand types, CO and cyclopentadienyl being two of them. Aromatic hydrocarbons constitute a further fundamental class of ligands, being isoelectronic with [C_5_H_5_]^–^ (Cp^–^) but providing a different hapticity and different coordinating properties. Whereas Cp^–^ is a ubiquitous ligand in organometallic chemistry, complexes with aromatic hydrocarbons C_6_H_*n*_R_6–*n*_ are much less explored, likely due to synthetic challenges and lower thermodynamic or kinetic stabilities. Since arene ligands are uncharged, vacancies at the metal center are more easily generated by haptotropic shifts than with Cp^–^. Binary arene complexes represent precursors for numerous reactions.^1–3^ After their discovery by Fischer and Hafner in 1955, a multitude of synthetic approaches were reported. A recent, excellent review comprehensively describes these synthetic strategies in detail.^4^ Of particular interest are syntheses starting from metal halides, in the absence or presence of reducing agents such as aluminum or zinc. Where metals such as Fe^II^ or Ru^II^ are already present in the desired oxidation state, no reducing agents are needed; higher starting oxidation states require reduction for coordination to the arenes.^5,6^

Bis-arene complexes [Re(η^6^-C_6_H_*n*_R_6–*n*_)_2_]^+^ (*n* = 1–6) are known but have been rarely studied and not in great detail. The first bis-arene complex of ^99^Tc was prepared by a very uncommon method, namely by element transmutation from [^99^Mo(arene)_2_] *via* β^–^-decay ^99^Mo → ^99m^Tc → ^99^Tc, the latter resulting directly from weighable amounts of ^99^TcCl_4_ in the presence of Al^0^ and AlCl_3_,^7,8^ in low yields.^9^ Only recently, Kudinov and coworkers reported an improved synthesis of [Re(arene)_2_]^+^ in moderate yields directly from K[ReO_4_] with Zn^0^ as the reductant, AlCl_3_ as a Lewis acid and the arene as solvent.^10^ For over 50 years, no progress was made in the study of [^99^Tc(η^6^-C_6_H_*n*_R_6–*n*_)_2_]^+^ type compounds, not only due to the radioactivity of this element but also because of synthetic difficulties. The preparation of binary halides such as ^99^TcCl_4_ as starting materials for accessing bis-arene complexes has made a significant impact over the past couple of years.^11–13^ Their syntheses are well described but their preparation and isolation are still not routine. A direct, high yield route to [^99^Tc(arene)_2_]^+^ complexes from [^99^TcO_4_]^–^ salts would therefore be highly desirable since it would allow for the chemical exploration of these fundamental complexes and for classifying their properties in relation to neighboring elements. In addition, arene complexes with metastable ^99m^Tc are potential molecular imaging agents: phenyl groups are frequent in pharmaceuticals and coordination of a ^99m^Tc fragment to such arenes enables labeling without additional, pendent ligands,^14^ a concept verified with the {Cr(CO)_3_} moiety bound to *e.g.* a phenyl ring in estradiol.^15,16^ The importance of the {M(arene)}^*n*+^ moiety is corroborated by the impact of the {Ru(cymantrene)}^2+^ fragment in medicinal inorganic chemistry.^17,18^ Among their endeavors for new myocardial imaging agents, Wester *et al.* reported the synthesis of [^99m^Tc(arene)_2_]^+^ complexes in the early nineties. However, since the yields were “variable”, the syntheses were multi-step, and the pharmacology was not satisfactory, the compounds were not studied any further.^19^ Enthusiastic about new ^99^Tc chemistry and convinced that the application of [^99m^Tc(arene)_2_]^+^ complexes will extend beyond myocardial imaging, we developed practical, one step syntheses of [^99(m)^Tc(arene)_2_]^+^ complexes in high yields and radiochemical purities, working towards a deeper understanding of the chemical and reactive properties of fundamental [^99^Tc(arene)_2_]^+^ complexes.

## Results and discussion

**Scheme 1:**
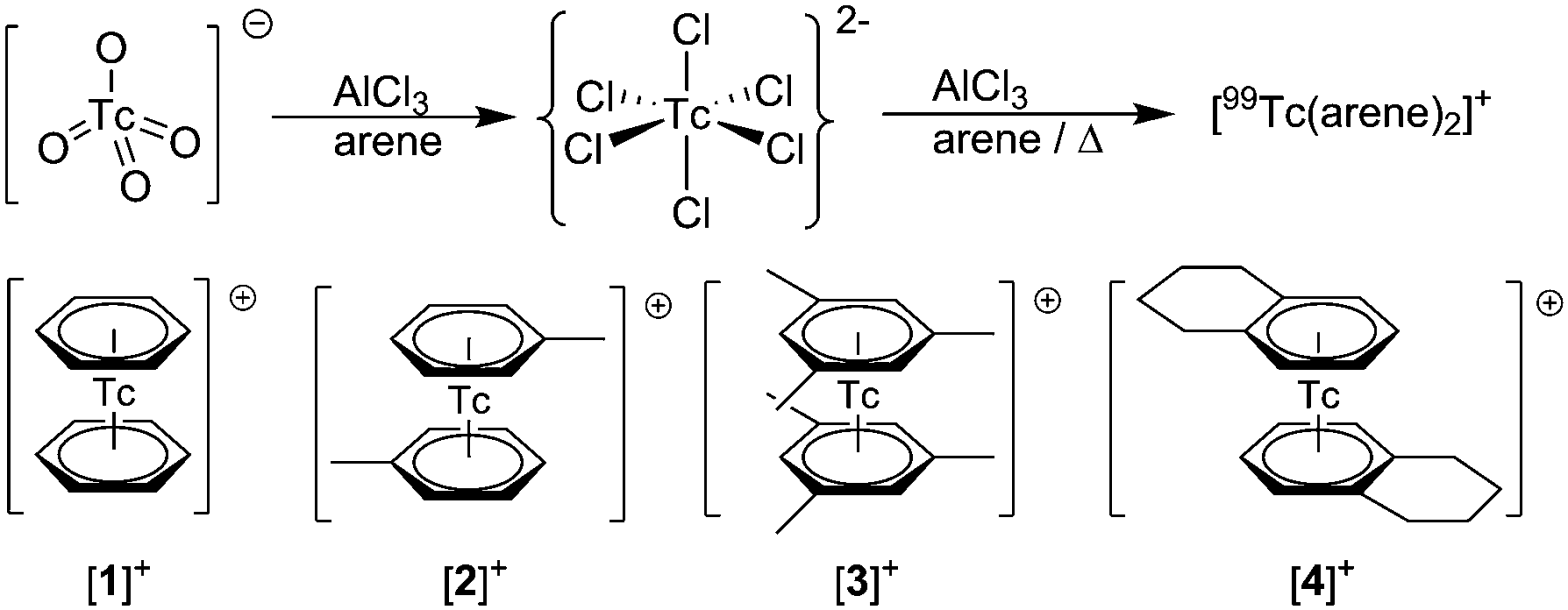
Reaction sequence for the preparation of [^99^Tc(arene)_2_]^+^ complexes.

The reaction of NH_4_[^99^TcO_4_], Zn^0^ and AlCl_3_ gave yields of <15% when applying reaction conditions analogous to those described for rhenium by Kudinov *et al.* The products were difficult to separate from a black, colloidal material (ESI, Section 1†). In contrast to classical Fischer–Hafner conditions, we carried out the reaction without Zn^0^, assuming that Zn^0^ could over-reduce the starting material to metallic, colloidal Tc^0^. Addition of toluene to solid AlCl_3_ and K[^99^TcO_4_] immediately produced a brown suspension. Heating the reaction mixture to 85 °C for 4 h gave a dark colored suspension. After extraction with water and precipitation with NH_4_PF_6_, we obtained [^99^Tc(C_6_H_5_CH_3_)_2_]^+^ as [**2**][PF_6_] in 75% isolated yield (Scheme 1). Comparable yields were obtained with mesitylene ([**3**][PF_6_]) and tetralin ([**4**][PF_6_]) (ESI, Section 1†). Only the reaction with benzene to afford [^99^Tc(C_6_H_6_)_2_]^+^ ([**1**][PF_6_]) required Zn^0^ (*vide infra*). With tetralin and AlCl_3_, we obtained product [**4**]^+^ but also a side product caused by Friedel–Crafts trans-alkylation reactions. Accordingly, the complex [^99^Tc(tetralin)(OHPhen)]^+^ (OHPhen = 1,2,3,4,5,6,7,8-octahydrophenanthrene) ([**5**][PF_6_]) was isolated and structurally characterised as a major side product of the reaction to form [**4**][PF_6_] (ESI Section 1 and 4†).

**Fig. 1:**
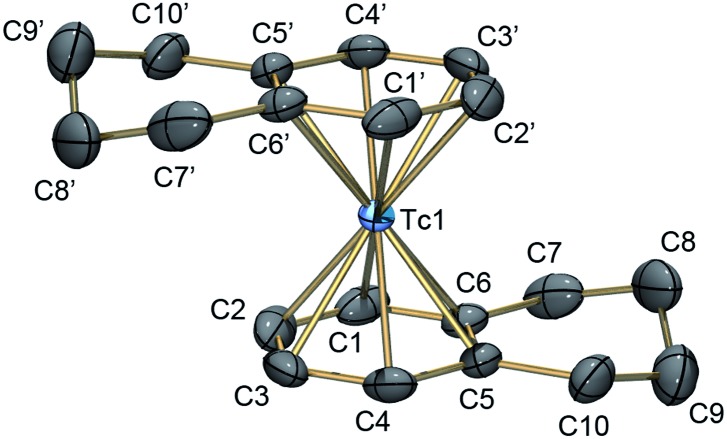
ORTEP representation^20^ of the [^99^Tc(tetralin)_2_]^+^ ([**4**]^+^) cation of the [**4**][PF_6_] structure. Thermal ellipsoids are represented at the 50% probability level. Hydrogen atoms are omitted for clarity. Selected bond lengths [Å] and angles [°]: Tc1–C1 2.219(2), Tc1–C2 2.228(2), Tc1–C3 2.231(2), Tc1–C4 2.227(2), Tc1–C5 2.250(2), Tc1–C6 2.250(2), C1–Tc1–C4 77.82(10), C2–Tc1–C5 78.34(9), C3–Tc1–C6 78.43(9).

To confirm the identities of [**1**][PF_6_], [**2**][PF_6_], [**3**][PF_6_] and [**4**][PF_6_], their structures were elucidated by single crystal X-ray diffraction (ESI, Section 4†). An ellipsoid representation of the tetralin complex is given in Fig. 1. These structures complete the series of bis-arene complexes with respect to the neighboring group 6 and 8 elements and the straight forward preparation paves the way for exploring fundamental chemistry and physico-chemical properties of [^99^Tc(η^6^-C_6_H_*n*_R_6–*n*_)_2_]^+^ type complexes.

We emphasise that no metallic reducing agent is present in these reactions. Whilst chloride reduces [^99^Tc^VII^O_4_]^–^ to Tc^V^ or Tc^IV^, arenes may also act as reductants under Friedel–Crafts conditions to achieve oxidation states lower than Tc^IV^. Indeed, when the reaction was carried out in cyclohexane and AlCl_3_ with [NBu_4_][^99^TcOCl_4_] and with no arene present, a yellow precipitate formed from a blue solution. Evaporation of the solvent and crystallization of the crude solid under N_2_ in dimethylformamide (DMF) gave large yellow crystals. From X-ray analysis the intermediate was confirmed to be [Al(DMF)_6_][^99^TcCl_6_]Cl·DMF. The presence of this Tc^IV^ complex supports the role of AlCl_3_ as a reducing agent of Tc^VII^ or Tc^V^to form Tc^IV^. Consequently, the reaction to afford [^99^Tc(arene)_2_]^+^ complexes can also be performed from K_2_[^99^TcCl_6_] with otherwise identical conditions as those from K[^99^TcO_4_] (ESI, Section 1†). The complexes [**2**][PF_6_] and [**4**][PF_6_] were prepared by this route in good yields.

These reactions represent a new procedure towards bis-arene complexes of group 7 elements. They combine pure arene substitution with elements already present in the desired oxidation state with concerted reduction–coordination, but without additional reductants such as Zn^0^ or Al^0^. Only one similar process, for [Fe(C_6_H_6_)_2_]^2+^, has been reported in the literature.^21^

The reaction of K[^99^TcO_4_] in benzene with AlCl_3_ did not lead to [^99^Tc(C_6_H_6_)_2_]^+^ ([**1**]^+^). In this reaction, only unidentified products formed and ^99^Tc NMR spectra gave no evidence for Tc^I^ species. Significant amounts of [^99^TcO_4_]^–^ (∼50%) could be recovered. We concluded that substituted alkenes with enhanced donating properties stabilised the intermediate oxidation states better than unsubstituted benzene. This interpretation is supported by the marked electrochemical differences found between the complexes (*vide infra*). Complex [**1**][PF_6_] could be synthesized from [^99^TcO_4_]^–^ *via* the Fischer–Hafner route in moderate yields with Zn dust as a reductant.

**Fig. 2:**
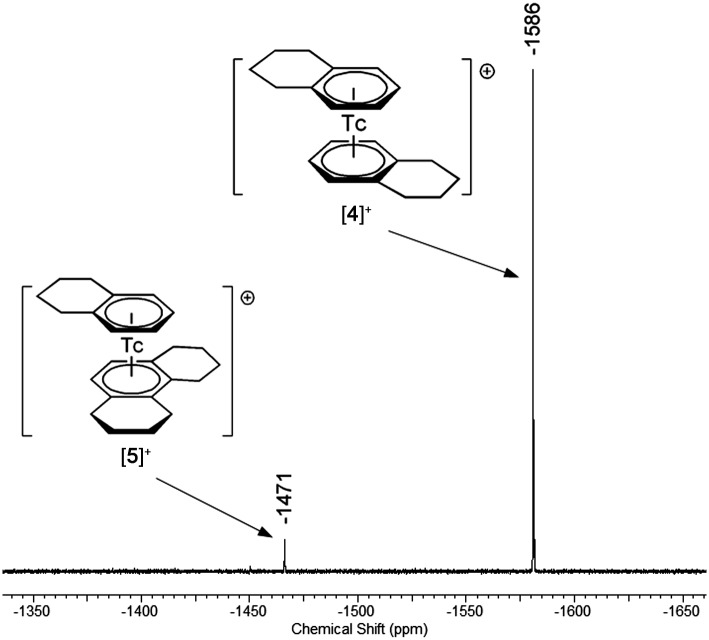
^99^Tc NMR spectrum of [^99^Tc(tetralin)_2_]^+^ (–1586 ppm, [**4**]^+^) and the side product [^99^Tc(tetralin)(OHPhen)]^+^ (–1471 ppm, [**5**]^+^).

^99^Tc NMR spectroscopy provides valuable insights into the symmetry and electronic properties of technetium complexes.^17,22^ Tc^I^ signals are typically found between –3000 and –1000 ppm relative to [^99^TcO_4_]^–^ at 0 ppm.^23^ Due to a scalar coupling of the nuclear spin (*I* = 9/2) to the quadrupole moment of the ^99^Tc nucleus, decreasing symmetry of the complexes is accompanied by a strong line broadening. However, the presented [^99^Tc(arene)_2_]^+^ complexes have highly symmetrical first coordination spheres, reflected in their small half line widths (8–26 Hz). The observed ^99^Tc NMR signals, [^99^Tc(benzene)_2_]^+^ at –1860 ppm (*ν*_1/2_ = 8 Hz), [^99^Tc(toluene)_2_]^+^ at –1744 ppm (*ν*_1/2_ = 26 Hz), [^99^Tc(tetralin)_2_]^+^ at –1586 ppm (*ν*_1/2_ = 11 Hz) and [^99^Tc(mesitylene)_2_]^+^ at –1532 ppm (*ν*_1/2_ = 14 Hz), suggest that a higher substitution pattern at the aromatic backbone leads to a shift in the ^99^Tc resonance to lower field. The ^99^Tc NMR spectrum of [**4**][PF_6_] (including the side product [**5**][PF_6_]) is shown in Fig. 2. All ^99^Tc NMR spectra are given in Section 3 of the ESI.† ^1^H NMR spectra show the strong influence of the metal center on the shift of the aromatic protons, which appear in the region around 5.5 ppm. Comparable features are found for isoelectronic complexes of the benzene complexes of Ru^II^ and Cr^0^, for which the arene signals are found at around 6.90 ^24^ and 4.21 ppm ^25^ respectively.

**Fig. 3:**
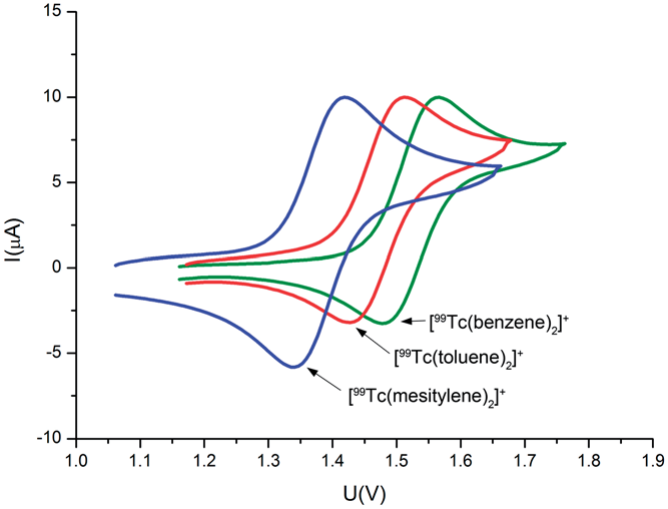
Cyclic voltammograms of [^99^Tc(benzene)_2_][PF_6_] (green line, *E*01/2 = 1.52 V), [^99^Tc(toluene)_2_][PF_6_] (red line, *E*01/2 = 1.47 V), and [^99^Tc(mesitylene)_2_][PF_6_] (blue line, *E*01/2 = 1.38 V) in acetonitrile *vs.* Fc/Fc^+^.

The small but significant chemical shift differences in the ^99^Tc NMR spectra of the complexes are caused by the different numbers of groups with negative Hammett constants. Increased electron donation to the ^99^Tc center should be paralleled with corresponding electrochemical properties. Cyclic voltammetry (CV) investigations are in agreement with this prediction and reversible oxidation waves for the Tc^I/II^ couple were found at surprisingly high values, generally *E*01/2 > +1.4 V *vs.* Fc/Fc^+^ in acetonitrile (Fig. 3 and ESI, Section 5†). Along the series [**1**]^+^ → [**4**]^+^, the oxidation potentials become less positive with an increasing number of donors on the arenes. Irreversible and essentially arene independent reductions assigned to the ^99^Tc^I/0^ couple appear below –2 V. As expected from general trends in the triads of the d-block elements, the rhenium homologues of [**1**]^+^ → [**4**]^+^ showed the Re^I/II^ couples shifted by about 0.19–0.15 V towards more negative potentials as compared to the ^99^Tc compounds (see ESI, Table ESI5.1.1†), *i.e.* they are more easily oxidised than the ^99^Tc homologues, albeit still at considerably positive potentials. Comparing the herein reported ^99^Tc potentials with those of the neighbouring elements, for those which are available, confirms general trends for d-block elements in the periodic system. For instance, *E*01/2 for [Cr(C_6_H_6_)_2_]^0/+^ is reported at +0.82 V *vs.* Ag/AgCl, and hence, although to our knowledge unreported, the corresponding molybdenum complex should have an even more negatively shifted potential. For [Ru(C_6_H_6_)_2_]^2+/3+^, no potentials for the Ru^II/III^ couple are reported, probably because observation of the oxidation is not possible in common solvents. On the other hand, *E*01/2 for the Ru^II/I^ couple is found at –1.02 V, almost 1 V more positive than the potential of its ^99^Tc analogue (Table ESI5.1.1†).^26–28^ Spectroelectrochemistry measurements in an OTTLE cell supported the reversibility of the [Re(arene)_2_]^+^/[Re(arene)_2_]^2+^ couple. The Re^I^ complex is oxidised to Re^II^ and reduced back to Re^I^ as is evident from the isosbestic points detected in the electrochemical experiment (Fig. ESI5.10.1†). In agreement with these electrochemical potentials, the [^99^Tc(arene)_2_]^+^ complexes are difficult to chemically oxidise and are very stable under ambient conditions. They are also insensitive to pH changes and do not decompose over the whole pH range even at elevated temperatures, an important feature for their potential application as molecular imaging agents.

**Scheme 2:**
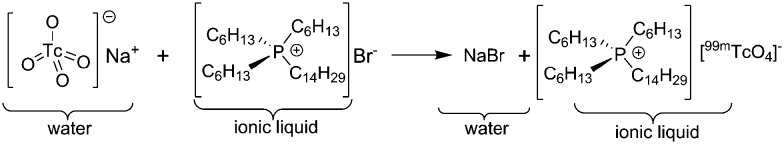
Extraction of Na[^99m^TcO_4_] from saline (0.9% NaCl in H_2_O) into an ionic liquid.

The high yields and one step syntheses to [^99^Tc(arene)_2_]^+^ complexes are the incentives for translating the reaction conditions to attain short-lived ^99m^Tc complexes. Apart from fundamental interests, conjugation of biologically active groups to the arene ring will open a path to novel, functionalised and targeted SPECT imaging agents. In the original preparation of [^99m^Tc(arene)_2_]^+^ complexes,^19^ the multiple steps required for transferring [^99m^TcO_4_]^–^ from the generator saline eluate into an organic solvent were difficult and time consuming, and ultimately prohibitive for any application. To facilitate this step, we coated a glass vial with a thin layer of ionic liquid (IL) by evaporating a dilute solution of [P(C_6_H_13_)_3_(C_14_H_29_)]Br in MTBE. Addition of the generator eluate to this vial resulted in extraction of 80–97% of [^99m^TcO_4_]^–^ into the IL layer within 10 min by an anion exchange process (Scheme 2). The saline was removed and the IL was dissolved in the corresponding arene and added to a vial charged with AlCl_3_ under N_2_. The vial was heated for 10 min at 100 °C in a microwave reactor. Upon addition of water, the [^99m^Tc(arene)_2_]^+^ complexes were extracted in yields of 35–87% and in radiochemical purities of >93%. Loading the aqueous solution onto a SepPak column and rinsing with water removed hydrolyzed AlCl_3_. The product was then eluted with an aqueous EtOH solution and isolated with >96% radiochemical purity (ESI, Section 1.2†).

The procedure was successfully applied for the toluene and mesitylene complexes. As described for [^99^Tc(tetralin)_2_]^+^, the yield of the [^99m^Tc(tetralin)_2_]^+^ complex was reduced due to formation of the trans-alkylated side product in a 1 : 0.8 ratio ([^99m^Tc(tetralin)_2_]^+^ : [^99m^Tc(tetralin)(OHPhen)]^+^). Comparable to the reactions with ^99^Tc, no additional reducing agent was required (except for the synthesis of [^99m^Tc(benzene)_2_]^+^). We emphasise that the ^99m^Tc bis-arene complexes are extraordinarily stable. Heating in an aqueous solution at 180 °C in a microwave oven in the presence of air does not decompose the complexes to a measurable extent. This stability against oxidation (and hydrolysis) is in agreement with the observed redox potentials and makes the complexes resistant to oxidation in air.

**Fig. 4:**
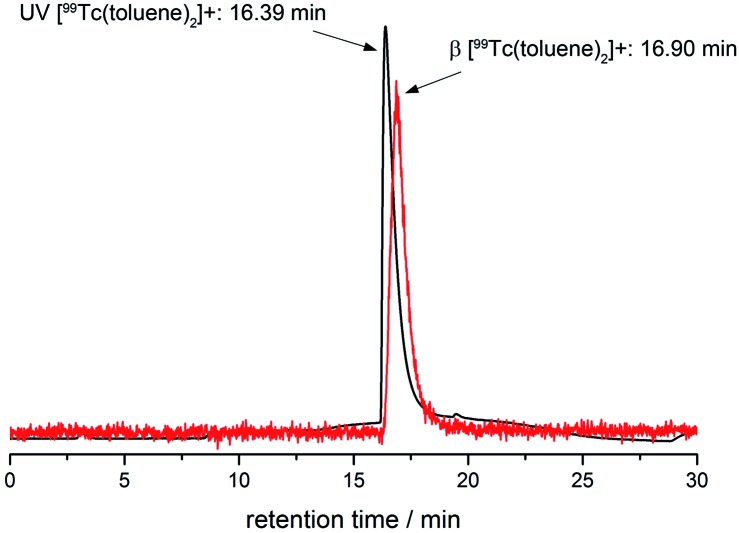
HPLC traces (UV-/β^–^-detection) of [^99^Tc(toluene)_2_]^+^ (UV-detection: 16.39 min, black line; β^–^-detection: 16.90 min, red line).

Assessment of the identities of the ^99m^Tc complexes by HPLC coinjection with fully characterised rhenium homologues and subsequent comparison of retention times is an FDA accepted procedure. Due to the serial arrangement of the UV- and radio-detectors, the respective γ-signal (^99m^Tc) is separated by a constant time delay (here 0.5 min) from the UV signal (Re). This time delay was quantified with [^99^Tc(toluene)_2_]^+^ (UV- and β^–^-detection for the same compound, Fig. 4).

Despite their isostructural nature and comparable physico-chemical properties, slightly different retention times (*R*_t_) (Δ*R*_t_ different from the 0.5 min) are sometimes observed for homologous ^99m^Tc and Re complexes. This makes their identification by *R*_t_ comparison somewhat ambiguous. The reported [^99m^Tc(arene)_2_]^+^ complexes provide good examples for this ambiguity, where the *R*_t_ for [^99m^Tc(benzene)_2_]^+^ is 0.58 min shorter than that of the corresponding Re complex. This effect diminishes with the increasing number of arene substituents ([^99m^Tc(toluene)_2_]^+^: 0.45 min, [^99m^Tc(mesitylene)_2_]^+^: 0.42 min, [^99m^Tc(tetralin)_2_]^+^: 0.39 min). These small but non-negligible uncertainties in the assessment of the identities of ^99m^Tc complexes can only be ruled out by HPLC coinjection with fully characterised ^99^Tc analogues; Δ*R*_t_ must be approximately 0.5 min (for our instrumental arrangement). This has additionally been verified by coinjection of [^99m^Tc(tetralin)_2_]^+^ and [^99^Tc(tetralin)_2_]^+^; the separation of the UV signal (^99^Tc complex) and the γ-signal (^99m^Tc) showed the expected separation (Δ*R*_t_ = 0.51 min, ESI, Section 2†). We emphasise that such direct comparisons between ^99m^Tc and ^99^Tc complexes are very rarely reported, if ever, but are ultimately the only way to truly assess the identity of a ^99m^Tc complex.

## Conclusions

Mono- and bis-arene complexes of d-block elements are fundamental in organometallic chemistry. The difficultly in accessing such complexes, especially for technetium where [^99^Tc(arene)_2_]^+^ type complexes have been essentially non-existent, has impeded their detailed chemical studies and their application in molecular imaging. We now present a new, high yielding synthetic procedure to afford these fundamental organometallic complexes of the group 7 elements. In our procedure no additional reducing agents are required, with the exception of the synthesis of [^99^Tc(benzene)_2_]^+^. AlCl_3_ serves as an oxygen abstracting Lewis acid and at the same time as a source of chloride reducing agent in the high valency regime. The reaction procedure can be fully applied to ^99m^Tc, enabling the introduction of novel organometallic complexes in the area of molecular imaging. Studies with non-alkyl arene substituents are currently ongoing.

## Experimental section

### General preparation of [^99^Tc(arene)_2_]^+^ complexes

**Caution**: ^99^Tc is a weak β^–^ emitter (*E*_max_ = 0.292 MeV, half life time = 2.12 × 10^5^ *y*. It should be handled only in appropriately equipped laboratories.

Method (a) NH_4_[^99^TcO_4_] (18 mg, 0.10 mmol), Zn-dust (22 mg, 0.34 mmol), AlCl_3_ (134 mg, 1.00 mmol) and the corresponding arene (6 ml) were heated to 85 °C. After 8 h, the solvent of the resulting dark brown suspension was removed with a stream of N_2_. The residue was washed with Et_2_O (3 × 2 ml). The remaining solid was extracted with H_2_O (3 × 2 ml) and the aqueous solution was filtered. NH_4_PF_6_ (150 mg, 0.92 mmol) in H_2_O (1 ml) was added to the red-brown filtrate. The colorless precipitate was filtered off, washed with H_2_O (2 × 0.5 ml) and Et_2_O (2 × 0.5 ml) and dried *in vacuo* to give [^99^Tc(arene)_2_](PF_6_) as a pale yellow powder. Alternatively, the precipitate can be extracted with CH_2_Cl_2_ from the aqueous suspension.

Method (b) K[^99^TcO_4_] (20 mg, 0.10 mmol), AlCl_3_ (200 mg, 1.50 mmol) and the corresponding arene (8 ml) were heated at 85 °C. After 4 h, H_2_O (6 ml) was added to the hot dark brown solution and the aqueous phase was separated and filtered. The process was repeated with additional H_2_O (2 × 2 ml). To the combined aqueous solutions was added a solution of NH_4_PF_6_ (150 mg, 0.92 mmol) in H_2_O (1 ml). The formed colorless precipitate was filtered, washed with H_2_O (2 × 0.5 ml) and dried *in vacuo* to give [^99^Tc(arene)_2_](PF_6_).

Method (c) K_2_[^99^TcCl_6_] (37 mg, 0.10 mmol), AlCl_3_ (134 mg, 1.00 mmol) and the corresponding arene (8 ml) were heated at 85 °C for 4 h. H_2_O (4 ml) was added to the hot dark brown solution and the aqueous phase was separated and filtered. The process was repeated with additional H_2_O (2 × 2 ml) and to the combined aqueous solutions was added a solution of NH_4_PF_6_ (150 mg, 0.92 mmol) in H_2_O (1 ml). The colorless precipitate was filtered, washed with H_2_O (2 × 0.5 ml) and dried *in vacuo* to give [^99^Tc(arene)_2_](PF_6_) as a pale yellow powder.

### General preparation of [^99m^Tc(arene)_2_]^+^ complexes

The ionic liquid [P(C_6_H_13_)_3_(C_14_H_29_)]Br (2 mg) dissolved in 0.1 ml methyl *tert*-butyl ether (MTBE) was added to a capped vial. Under constant rotation of the vial, the solvent was evaporated by a N_2_ stream. The [^99m^TcO_4_]^–^ eluate (1–2 ml) was added and the vial was gently shaken for 10 min. The aqueous solution was removed and the vial was purged with N_2_ for 30 min. 80–97% of the [^99m^TcO_4_]^–^ remained in the vial. The ionic liquid was dissolved in the corresponding arene (1 ml). This solution was added to a vial charged with AlCl_3_ (100 mg) under N_2_. The reaction mixture was heated for 10 min at 100 °C in a microwave oven. 1 ml of saline solution was added to the yellow reaction mixture and the vial was vortexed for 20 s and centrifuged for 6 min. The aqueous phase was separated with a syringe and contained >90% of the final product. The workup procedure was repeated for a second time. Standard SepPack procedure removed salts and the product was eluted with ethanol.
