# Supplementary material for: From TcVII to TcI; facile syntheses of bis-arene complexes [99(m)Tc(arene)2]+ from pertechnetate
Source: Chem Sci. 2014 Sep 5;6(1):165–9. doi: 10.1039/c4sc02461c (PMC5424581; doi:10.1039/c4sc02461c)
Supplement: Supplementary file 1 [file SC-006-C4SC02461C-s001.pdf]

Electronic Supplementary Material (ESI) for Chemical Science.
This journal is © The Royal Society of Chemistry 2014

From Tc^{VII} to Tc^{I}; Facile Syntheses of Bis-arene Complexes $[^{99(m)}Tc(arene)_2]^+$ from Pertechnetate

Michael Benz, Henrik Braband, Paul Schmutz, Jonathan Halter and Roger Alberto

Department of Chemistry, University of Zürich, Winterthurerstr. 190, CH-8057 Zürich Switzerland

Supporting Information

Table of Contents

1 Experimental Section

1.1 General procedures for the preparation of $[^{99}Tc(arene)_2](PF_6)$
- 1.1.1 $[^{99}Tc(benzene)_2](PF_6)$ ([**1**](PF_6))
- 1.1.2 $[^{99}Tc(toluene)_2](PF_6)$ ([**2**](PF_6))
- 1.1.3 $[^{99}Tc(mesitylene)_2](PF_6)$ ([**3**](PF_6))
- 1.1.4 $[^{99}Tc(tetralin)_2](PF_6)$ ([**4**](PF_6))

1.2 General procedures for the preparation of $[^{99m}Tc(arene)_2]^+$ complexes
- 1.2.1 $[^{99m}Tc(benzene)_2]^+$ ($[^{99m}\mathbf{1}]^+$)
- 1.2.2 $[^{99m}Tc(toluene)_2]^+$ ($[^{99m}\mathbf{2}]^+$)
- 1.2.3 $[^{99}Tc(mesitylene)_2]^+$ ($[^{99m}\mathbf{3}]^+$)
- 1.2.4 $[^{99}Tc(tetralin)_2]^+$ ($[^{99m}\mathbf{4}]^+$)

2 HPLC Data

- 2.1 Coinjection of $[^{99m}Tc(benzene)_2]^+$ with $[Re(benzene)_2]^+$
- 2.2 Coinjection of $[^{99m}Tc(toluene)_2]^+$ with $[Re(toluene)_2]^+$
- 2.3 Coinjection of $[^{99m}Tc(toluene)_2]^+$ with $[^{99}Tc(toluene)_2]^+$
- 2.4 $[^{99m}Tc(toluene)_2]^+$ yields vs. amount $AlCl_3$
- 2.5 Coinjection of $[^{99m}Tc(mesitylene)_2]^+$ with $[Re(mesitylene)_2]^+$
- 2.6 Coinjection of $[^{99m}Tc(tetralin)_2]^+$ with $[Re(tetralin)_2]^+$
- 2.7 Coinjection of $[^{99m}Tc(tetralin)_2]^+$ with $[^{99}Tc(tetralin)_2]^+$

3 NMR Data

3.1 Overview NMR Data

3.2 $[^{99}Tc(benzene)_2](PF_6)$ ([**1**](PF_6))
- 3.2.1 ^{1}H NMR $[^{99}Tc(benzene)_2](PF_6)$
- 3.2.2 ^{13}C NMR $[^{99}Tc(benzene)_2](PF_6)$
- 3.2.3 ^{99}Tc NMR $[^{99}Tc(benzene)_2](PF_6)$

3.3 $[^{99}Tc(toluene)_2](PF_6)$ ([**2**](PF_6))
- 3.3.1 ^{1}H NMR $[^{99}Tc(toluene)_2](PF_6)$
- 3.3.2 ^{13}C NMR $[^{99}Tc(toluene)_2](PF_6)$
- 3.3.3 ^{99}Tc NMR $[^{99}Tc(toluene)_2](PF_6)$

3.4 [^{99}Tc(mesitylene)$_2$](PF$_6$) ([**3**](PF$_6$))
3.4.1 ^{1}H NMR [^{99}Tc(mesitylene)$_2$](PF$_6$)
3.4.2 ^{13}C NMR [^{99}Tc(mesitylene)$_2$](PF$_6$)
3.4.3 ^{99}Tc NMR [^{99}Tc(mesitylene)$_2$](PF$_6$)
3.5 [^{99}Tc(tetralin)$_2$](PF$_6$) ([**4**](PF$_6$))
3.5.1 ^{1}H NMR [^{99}Tc(tetralin)$_2$](PF$_6$)
3.5.2 ^{13}C NMR [^{99}Tc(tetralin)$_2$](PF$_6$)
3.5.3 ^{99}Tc NMR [^{99}Tc(tetralin)$_2$](PF$_6$)

4 Crystallographic Details

4.1 [^{99}Tc(benzene)$_2$](PF$_6$) ([**1**](PF$_6$))
4.2 [^{99}Tc(toluene)$_2$](PF$_6$) ([**2**](PF$_6$))
4.3 [^{99}Tc(mesitylene)$_2$](PF$_6$) ([**3**](PF$_6$))
4.4 [^{99}Tc(tetralin)$_2$](PF$_6$) ([**4**](PF$_6$))
4.5 [^{99}Tc(tetralin)(OHPhen)]$^+$ ([**5**]$^+$)
4.6 [Al(dmf)$_6$][^{99}TcCl$_6$]Cl·(dmf)

5 CV Data

5.1 Overview CV Data
5.2 [^{99}Tc(benzene)$_2$](PF$_6$) ([**1**](PF$_6$))
5.3 [^{99}Tc(toluene)$_2$](PF$_6$) ([**2**](PF$_6$))
5.4 [^{99}Tc(mesitylene)$_2$](PF$_6$) ([**3**](PF$_6$))
5.5 [^{99}Tc(tetralin)$_2$](PF$_6$) ([**4**](PF$_6$))
5.6 [Re(benzene)$_2$](PF$_6$)
5.7 [Re(toluene)$_2$](OTf)
5.8 [Re(mesitylene)$_2$](PF$_6$)
5.9 [^{99}Tc(tetralin)$_2$](OTf)
5.10 Spectroelectrochemistry (OTTEL cell)

1 Experimental Section

General Information

Caution: ^{99}Tc is a weak β^- emitter. All experiments have to be done in laboratories approved for working with low-level radioactive materials.

All reactions were carried out under an inert N_2 atmosphere. $(NH_4)[^{99}TcO_4]$ (Oak Ridge) and all other chemicals were of reagent grade and used without further purification.
FT-IR spectra were measured as KBr pellets on a Perkin Elmer Spectrum Two spectrophotometer. ^1H and ^{13}C NMR spectra were recorded on a Bruker DRX500 500 MHz or a Bruker AV-400 400 MHz spectrometer. ^{13}C NMR spectra were proton decoupled recorded. For technetium content measurements, pure compounds were dissolved in the appropriate solvents. The measurements were carried out with a scintillation cocktail (Packard Ultimate Gold XR) and a liquid scintillation counter (TRICARB 2200CA, Packard). HPLC analyses were performed on a Merck Hitachi LaChrom L 7100 pump coupled to a Merck Hitachi LaChrom L7200 tunable UV detector and a radiodetector. UV/Vis detection was performed at 250 nm. The detection of radioactive $^{99}Tc/^{99m}Tc$ complexes was performed with a Berthold LB508 radiodetector equipped with YG/BGO cells, respectively. Separations were achieved on a Macherey-Nagel C18 reversed-phase column (Nucleosil 10 lm, 250 4 mm) using a gradient of MeOH/ 0.1% CF_3COOH as eluent, and a flow rate of 0.5 mL/min. Gradient: t = 0 – 3 min: 0% MeOH; 3 – 3.1 min: 0 – 25% MeOH; 3.1 – 9 min: 25% MeOH; 9 – 9.1 min: 25 – 34% MeOH; 9.1 – 18 min: 34 - 100% MeOH; 18 – 25 min: 100% MeOH, 25 – 25.1 min: 100 - 0% MeOH; 25.1 – 30 min: 0% MeOH. Comparison of the HPLC retention times for the ^{99m}Tc compounds with the corresponding ^{99}Tc and Re compounds confirms identity. Electrochemical measurements were carried out in acetonitrile containing 0.1 M $[TBA][PF_6]$ as a conducting electrolyte. For all Tc-Complexes, a Metrohm 757VA Computrace electrochemical analyzer was used with a standard three-electrode setup of glassy carbon working electrode (i.d.= 3 mm), platinum auxiliary electrode and Ag/AgCl reference electrode. For all Re-Complexes, a Metrohm 797VA Computrace electrochemical analyzer was used with the same three-electrode setup. All potential are given vs Ag/AgCl and are referenced with Fc/Fc+ at 450 mV. Spectroelectrochemical analysis were carried out in MeCN containing 0.1 M $[TBA][PF_6]$ as conducting electrolyte. The measurements were performed in a 5 mm wide optical transparent thin-layer electrolysis (OTTLE) cell in a Cary 50 spectrometer for UV/VIS detection. The working electrode was a platinum gaze immersed into the OTTLE cell, auxiliary electrode was a platinum wire and the reference electrode was an Ag/AgCl electrode.

1.1 General procedures for the preparation of $[^{99}Tc(arene)_2](PF_6)$

Method a) $(NH_4)[^{99}TcO_4]$ (18 mg, 0.10 mmol), Zn-dust (22 mg, 0.34 mmol), $AlCl_3$ (134 mg, 1.00 mmol) and the corresponding arene (6 ml) were heated to 85°C. After 8 h, the solvent of the resulting dark brown suspension was removed with a stream of N_2. The residue was washed with Et_2O (3 × 2 ml). The remaining solid was extracted with H_2O (3 × 2 ml) and the aqueous solution filtered. NH_4PF_6 (150 mg, 0.92 mmol) in H_2O (1 ml) was added to the red-brown filtrate. The colorless precipitate was filtered off, washed with H_2O (2 × 0.5 ml) and Et_2O (2 × 0.5 ml) and dried *in vacuo* to give $[^{99}Tc(arene)_2](PF_6)$ as a pale yellow powder. Alternatively, the precipitate can be extracted by CH_2Cl_2 from the aqueous suspension. Crystals, suitable for X-ray diffraction analysis were grown by slow diffusion of Et_2O into an acetone solution of [**1-4**](PF_6).

Method b) $K[^{99}TcO_4]$ (20 mg, 0.10 mmol), $AlCl_3$ (200 mg, 1.50 mmol) and the corresponding arene (8 ml) were heated at 85°C. After 4 h, H_2O (6 ml) was added to the hot dark brown solution and the

aqueous phase was separated and filtered. The process was repeated with additional H_2O (2 × 2 ml). To the combined aqueous solutions was added a solution of NH_4PF_6 (150 mg, 0.92 mmol) in H_2O (1 ml). The formed colorless precipitate was filtered, washed with H_2O (2 × 0.5 ml) and dried *in vacuo* to give [Tc(toluene)$_2$](PF_6) as a pale yellow powder.
Method c) $K_2[^{99}TcCl_6]$ (37 mg, 0.10 mmol), $AlCl_3$ (134 mg, 1.00 mmol) and the corresponding arene (8 ml) were heated on 85°C for 4 h. H_2O (4 ml) was added to the dark brown hot solution and the aqueous phase was separated and filtered. The process was repeated with additional H_2O (2 × 2 ml) and to the combined aqueous solution was added a solution of NH_4PF_6 (150 mg, 0.92 mmol) in H_2O (1 ml). The formed colorless precipitate was filtered, washed with H_2O (2 × 0.5 ml) and dried *in vacuo* to give [Tc(arene)$_2$](PF_6) as a pale yellow powder.

1.1.1 [^{99}Tc(benzene)$_2$](PF_6) ([**1**](PF_6))
Method a) Yield: 3 mg (8%).
IR (KBr): 3435 (*m*), 3095 (*w*), 2920 (*w*), 2851 (*w*), 1630 (*w*), 1441 (*m*), 1387 (*w*), 1156 (*w*), 1024 (*w*), 922 (*w*), 836 (*s*), 820 (*s*), 557 (*m*), 430 (*m*) cm^{-1}.^{1}H NMR (400 MHz, MeCN-d$_3$): δ = 5.65 (s, 12 H, C$H_{arom.}$) ppm. ^{13}C NMR (125 MHz, MeCN-d$_3$): δ = 85.05-82.04 (m, CH$_{arom.}$) ppm. ^{99}Tc NMR (90 MHz, MeCN-d$_3$): δ = -1860 (*s*, $\Delta\nu_{1/2}$ = 8 Hz) ppm. ^{99}Tc analysis: calcd. 24.74%; found 24.23%.

1.1.2 [^{99}Tc(toluene)$_2$](PF_6) ([**2**](PF_6))
Method a) Yield: 3 mg (7%).
Method b) Yield: 32 mg (75%).
Method c) Yield: 25 mg (60%).
IR (KBr): 3435 (*m*), 3090 (*w*), 2919 (*w*), 2851 (*w*), 1634 (*w*), 1574 (*w*), 1514 (*w*), 1455 (*m*), 1388 (*w*), 1043 (*w*), 835 (*s*), 777 (*w*), 558 (*m*), 441 (*w*), 410 (*w*) cm^{-1}. ^{1}H NMR (500 MHz, MeCN-d$_3$): δ = 5.63 (s, 4 H, C$H_{arom.}$), 5.55 (s, 4 H, C$H_{arom.}$), 5.50 (s, 2 H, C$H_{arom.}$), 2.12 (s, 6 H, CH_3) ppm. ^{13}C NMR (125 MHz, MeCN-d$_3$): δ = 87.26-81.43 (m, CH$_{arom.}$), 20.16 (s, CH$_3$) ppm. ^{99}Tc NMR (90 MHz, MeCN-d$_3$): δ = -1744 (*s*, $\Delta\nu_{1/2}$ = 26 Hz) ppm. ^{99}Tc analysis: calcd. 23.11%; found 22.71%.

1.1.3 [^{99}Tc(mesitylene)$_2$](PF_6) ([**3**](PF_6))
Method a) Yield: 6 mg (12%).
Method b) Yield: 43 mg (90%).
IR (KBr): 3435 (*m*), 3086 (*w*), 2988 (*w*), 2967 (*w*), 2923 (*w*), 2847 (*w*), 1630 (*w*), 1540 (*w*), 1458 (*m*), 1384 (*w*), 1039 (*m*), 908 (*w*), 874 (*w*), 836 (*s*), 558 (*m*), 515 (*w*), 417 (*w*) cm^{-1}. ^{1}H NMR (400 MHz, MeCN-d$_3$): δ = 5.39 (s, 6 H, C$H_{arom.}$), 2.04 (s, 9 H, CH_3) ppm. ^{13}C NMR (125 MHz, MeCN-d$_3$): δ = 99.86-96.64 (m, ^{t}CH$_{arom.}$), 87.94-84.70 (m, CH$_{arom.}$), 19.33 (s, CH$_3$) ppm. ^{99}Tc NMR (90 MHz, MeCN-d$_3$): δ = -1532 (*s*, $\Delta\nu_{1/2}$ = 14 Hz) ppm. ^{99}Tc analysis: calcd. 20.44%; found 20.39%.

1.1.4 [^{99}Tc(tetralin)$_2$](PF_6) ([**4**](PF_6))
Method a) Yield: 4 mg (8%).
Method b) Yield: 34 mg (69%).
Method c) Yield: 30 mg (63%).
IR (KBr): 3430 (*m*), 3086 (*w*), 2948 (*s*), 2869 (*m*), 1630 (*w*), 1484 (*w*), 1457 (*s*), 1435 (*s*), 1413 (*w*), 1357 (*w*), 1340 (*w*), 1282 (w), 1251 (*w*), 1152 (*w*), 1088 (*w*), 1071 (*w*), 1015 (*w*), 990 (*w*), 921 (*w*), 909 (*m*), 833 (*s*), 795 (*m*), 708 (*w*), 596 (*w*), 557 (*s*), 473 (*w*), 409 (*w*) cm^{-1}. ^{1}H NMR (500 MHz, MeCN-d$_3$): δ = 5.54 (s, 4 H, C$H_{arom.}$), 5.43 (s, 4 H, C$H_{arom.}$), 2.77-2.51 (m, CH_2), 1.73 (s, 4 H, CH_2) ppm. ^{13}C NMR (125 MHz, MeCN-d$_3$): δ = 103.12-100.16 (m, ^{t}CH$_{arom.}$), 85.98-82.33 (m, CH$_{arom.}$), 28.35 (CH$_2$), 22.96 (CH$_2$) ppm. ^{99}Tc NMR (90 MHz, MeCN-d$_3$): δ = -1586 (*s*, $\Delta\nu_{1/2}$ = 11 Hz) ^{99}Tc analysis: calcd. 19.47%; found 18.73%.

$[^{99}Tc(tetralin)(OHPhen)]^+$ (OHPhen = 1,2,3,4,5,6,7,8-octahydrophenanthrene, [**5**](PF_6))
The isolated product [**4**](PF_6) contained very small amounts of an unknown impurity (s. ^{99}Tc NMR figure ESI3.5.3.1). After crystallization of the product few crystals of different shapes could be identified, collected and analyzed. In this way the minor side product $[^{99}Tc(tetralin)(OHPhen)]^+$ (OHPhen = 1,2,3,4,5,6,7,8-octahydrophenanthrene) has been characterized by NMR, ESI MS, and X-ray diffraction.
^{99}Tc NMR (90 MHz, MeCN-d_3): δ = -1470 (*s*, $\Delta\nu_{1/2}$ = 21 Hz)
ESI^+ MS: *m/z*= 417.2 (M^+)

1.2 General procedures for the preparation of $[^{99m}Tc(arene)_2]^+$ complexes

Trihexyl(tetradecyl)phosphonium bromide (2 mg) dissolved in 0.1 ml methyl tert-butyl ether (MTBE) was added to a capped vial. Under constant rotation of the vial, the solvent was evaporated by a nitrogen stream. The $[^{99m}TcO_4]^-$ eluate (1 -2 ml) was added to this vial and gently shaken for 10 min. The aqueous solution was removed and the vial was purged with nitrogen gas for 30 min. These prepared vials contained 80 - 97% of the starting activity. The ionic liquid was dissolved in the corresponding arene (1 ml) and the solution was added to a vial, charged with $AlCl_3$ (100mg) under a nitrogen gas atmosphere. For the $[^{99m}Tc(benzene)_2]^+$ synthesis Zn-dust (6 mg) has been added to the $AlCl_3$. By means of an microwave reactor, the reaction mixture was heated for 10 min at 100°C. Afterwards, 1 ml of saline solution was added to the yellow reaction mixture. The vial was vortexed for 20 sec. and centrifuged for 6 min. The aqueous phase was separated with a syringe. The aqueous phase contained > 90% of the final product. However, the workup procedure was repeated for a second time. Finally, the product was formulated by a standard SepPack procedure.

1.2.1 $[^{99m}Tc(benzene)_2]^+$ ($[^{99m}\mathbf{1}]^+$)
Yield after reaction: 87% (99% radiochemical pure)
Yield after formulation: 67% (> 99.9% radiochemical pure)

1.2.2 $[^{99m}Tc(toluene)_2]^+$ ($[^{99m}\mathbf{2}]^+$)
Yield after reaction: 80% (> 99.9% radiochemical pure)
Yield after formulation: 58% (> 99.9% radiochemical pure)

1.2.3 $[^{99m}Tc(mesitylene)_2]^+$ ($[^{99m}\mathbf{3}]^+$)
Yield after reaction: 54%(> 93% radiochemical pure)
Yield after formulation: 43% (> 96 % radiochemical pure)

1.2.4 $[^{99m}Tc(tetralin)_2]^+$ ($[^{99m}\mathbf{4}]^+$)
Yield after reaction: 35% (21% radiochemical pure)
Yield after formulation: 15.4% (48% radiochemical pure)

2 HPLC Data

2.1 Coinjection of [^{99m}Tc(benzene)$_2$]$^+$ with [Re(benzene)$_2$]$^+$

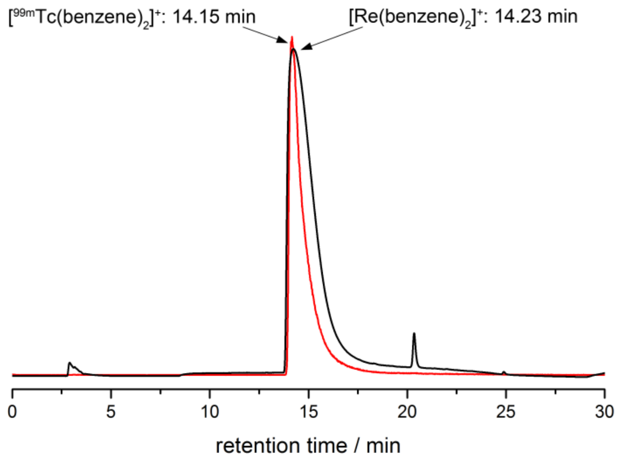

Figure ESI2.1: HPLC traces (UV- / γ-detection) of the coinjection of [^{99m}Tc(benzene)$_2$]$^+$ (14.15 min, red line) and [Re(benzene)$_2$]$^+$ (14.23 min, black line).

2.2 Coinjection of [^{99m}Tc(toluene)$_2$]$^+$ with [Re(toluene)$_2$]$^+$

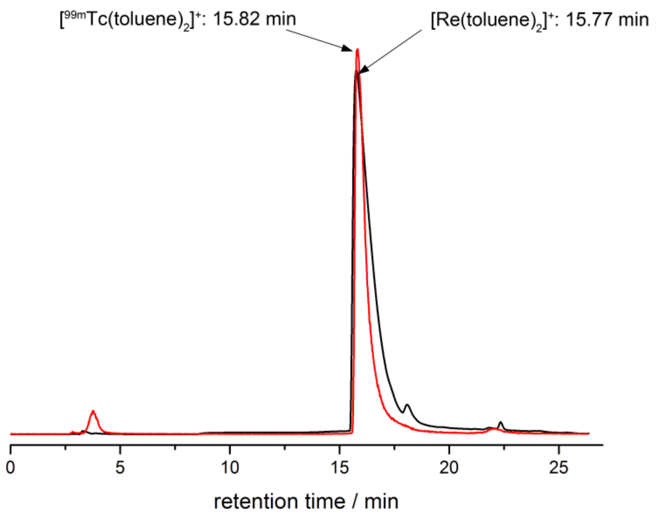

Figure ESI2.2: HPLC traces (UV- / γ-detection) of the coinjection of [^{99m}Tc(toluene)$_2$]$^+$ (15.82 min, red line) and [Re(toluene)$_2$]$^+$ (15.77 min, black line).

2.3 Coinjection of [^{99m}Tc(toluene)$_2$]$^+$ with [^{99}Tc(toluene)$_2$]$^+$

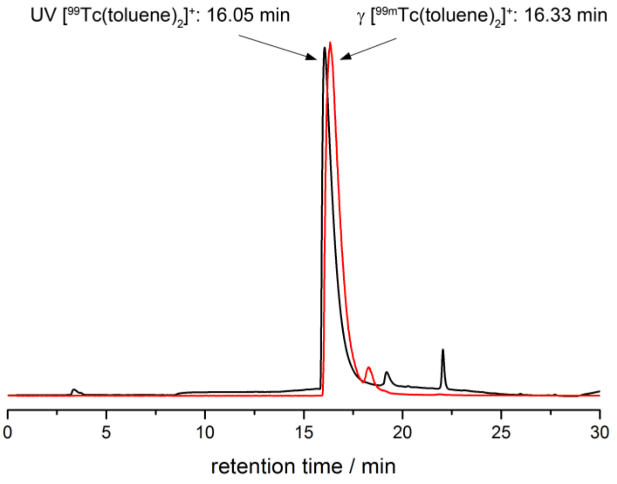

Figure ESI2.3: HPLC traces (UV- / γ-detection) of the coinjection of [^{99m}Tc(toluene)$_2$]$^+$ (16.33 min, red line) and [^{99}Tc(toluene)$_2$]$^+$ (16.05 min, black line).

2.4 [^{99m}Tc(toluene)$_2$]$^+$ yields vs. amount $AlCl_3$

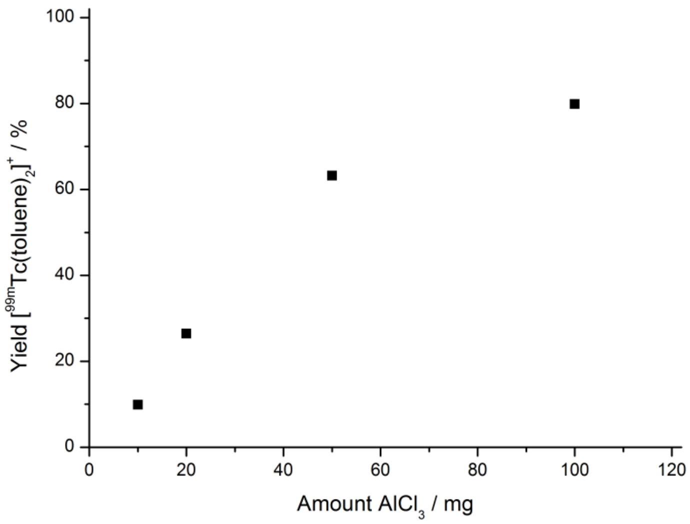

Figure ESI2.4: [^{99m}Tc(toluene)$_2$]$^+$ yields vs. amount $AlCl_3$

2.5 Coinjection of [^{99m}Tc(mesitylene)$_2$]$^+$ with [Re(mesitylene)$_2$]$^+$

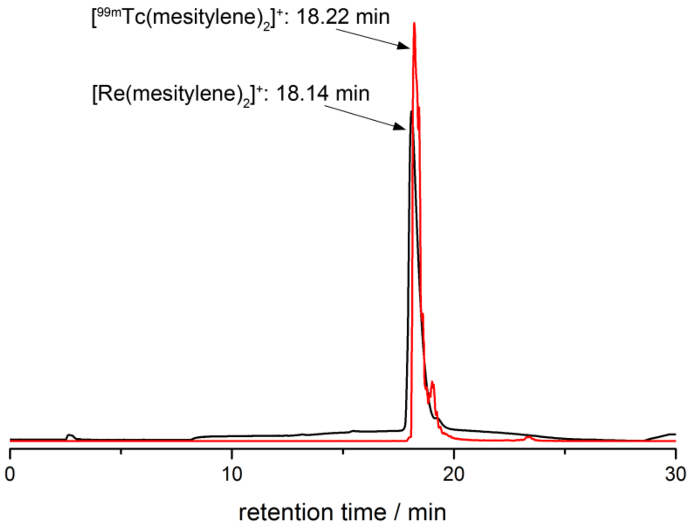

Figure ESI2.5: HPLC traces (UV- / γ-detection) of the coinjection of [^{99m}Tc(mesitylene)$_2$]$^+$ (18.22 min, red line) and [Re(mesitylene)$_2$]$^+$ (18.14 min, black line).

2.6 Coinjection of [^{99m}Tc(tetralin)$_2$]$^+$ with [Re(tetralin)$_2$]$^+$

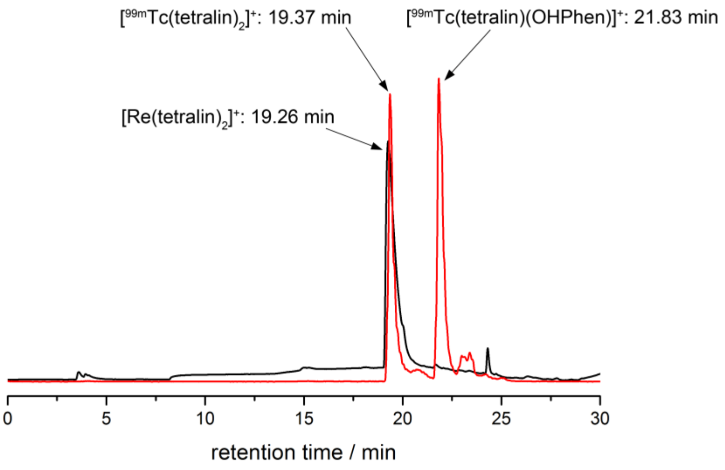

Figure ESI2.6: HPLC traces (UV- / γ-detection) of the coinjection of [^{99m}Tc(teralin)$_2$]$^+$ (19.37 min, red line) and [Re(tetralin)$_2$]$^+$ (19.26 min, black line). The peak at 21.83 min (red line) represents the side product [^{99m}Tc(teralin)(OHPhen)]$^+$ (OHPhen = 1,2,3,4,5,6,7,8-octahydrophenanthrene).

2.7 Coinjection of $[^{99m}Tc(tetralin)_2]^+$ with $[^{99}Tc(tetralin)_2]^+$

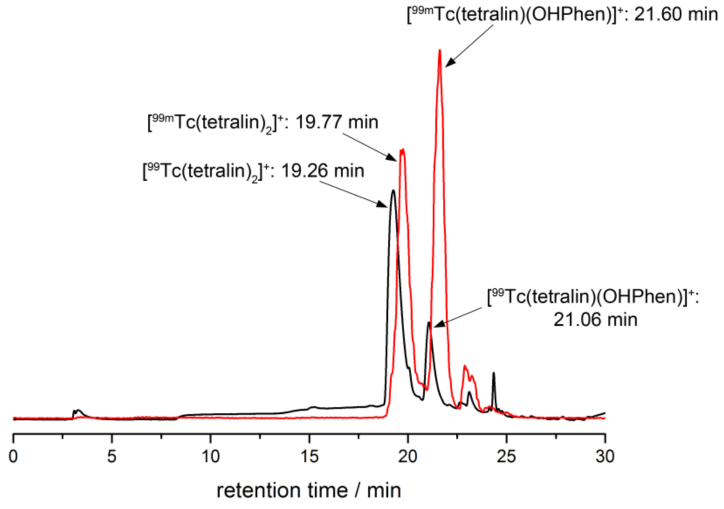

Figure ESI2.7: HPLC traces (UV- / γ-detection) of the coinjection of $[^{99m}Tc(teralin)_2]^+$ (19.77 min, red line) and $[^{99}Tc(tetralin)_2]^+$ (19.26 min, black line). The peaks at 21.60 min (red line) and 21.06 min (black line) represent the side products $[M(teralin)(OHPhen)]^+$ (M = ^{99m}Tc, ^{99}Tc; OHPhen = 1,2,3,4,5,6,7,8-octahydrophenanthrene) respectively.

3 NMR Data

3.1 Overview NMR Data

Table ESI3.1.1: Overview ^{1}H-NMR-data

Compound	δ C-H [ppm]	δ CH_3/CH_2 [ppm]
[^{99}Tc(benzene)$_2$](PF_6)[a, 1]	5.65	-
[^{99}Tc(toluene)$_2$](PF_6)[a, 1]	5.63, 5.55, 5.50	2.12
[^{99}Tc(mesitylene)$_2$](PF_6)[a, 1]	5.39	2.04
[^{99}Tc(tetralin)$_2$](PF_6)[a, 1]	5.54, 5.43	2.77-2.51, 1.73
[Re(benzene)$_2$](PF_6)[b,5]	6.14	-
[Re(toluene)$_2$](PF_6)[b, 5]	6.16, 6.06, 6.00	2.32
[Fe(benzene)$_2$](PF_6)$_2$[a, 6]	6.94	-
[Fe(toluene)$_2$](PF_6)$_2$[a, 6]	6.85, 6.79	2.56
[Fe(mesitylene)$_2$](PF_6)$_2$[a, 6]	6.44	2.53
[Ru(benzene)$_2$](PF_6)$_2$[c, 7]	6.90	-
[Ru(C_6Me_6)$_2$](PF_6)$_2$[b, 7]	-	2.35
[Os(C_6Me_6)$_2$](BF_4)$_2$[c, 8]	-	2.15
[Cr(benzene)$_2$][d, 9]	4.21	-
[Mo(benzene)$_2$][d, 10]	4.60	-
[Mo(toluene)$_2$][d, 10]	4.60	1.89
[W(benzene)$_2$][d, 10]	5.00	-
[W(toluene)$_2$][d, 10]	4.90	1.97

[a] in CD_3CN; [b] in acetone-d6; [c] in DMSO-d_6; [d] in perdeuteriobenzene or toluene;

Table ESI3.1.2: Overview ^{13}C-NMR-data

Compound	δ M-C[ppm]	δ CH_3/CH_2 [ppm]
[^{99}Tc(benzene)$_2$](PF_6)[a, 1]	85.05-82.04	-
[^{99}Tc(toluene)$_2$](PF_6)[a, 1]	87.26-81.43	20.16

$[^{99}Tc(mesitylene)_2](PF_6)$[a, 1]	99.86-96.64, 87.94-84.70	19.33
$[^{99}Tc(tetralin)_2](PF_6)$[a, 1]	103.12-100.16, 85.98-82.33	28.35, 22.96
$[Os(C_6Me_6)_2](BF_4)_2$[c, 9]	100.31	15.15

[a] in CD_3CN; [b] in acetone-d6; [c] in DMSO-d_6; [d] in perdeuteriobenzene or toluene;

3.2 [^{99}Tc(benzene)$_2$](PF$_6$) ([1](PF$_6$))

3.2.1 ^{1}H NMR [^{99}Tc(benzene)$_2$](PF$_6$)

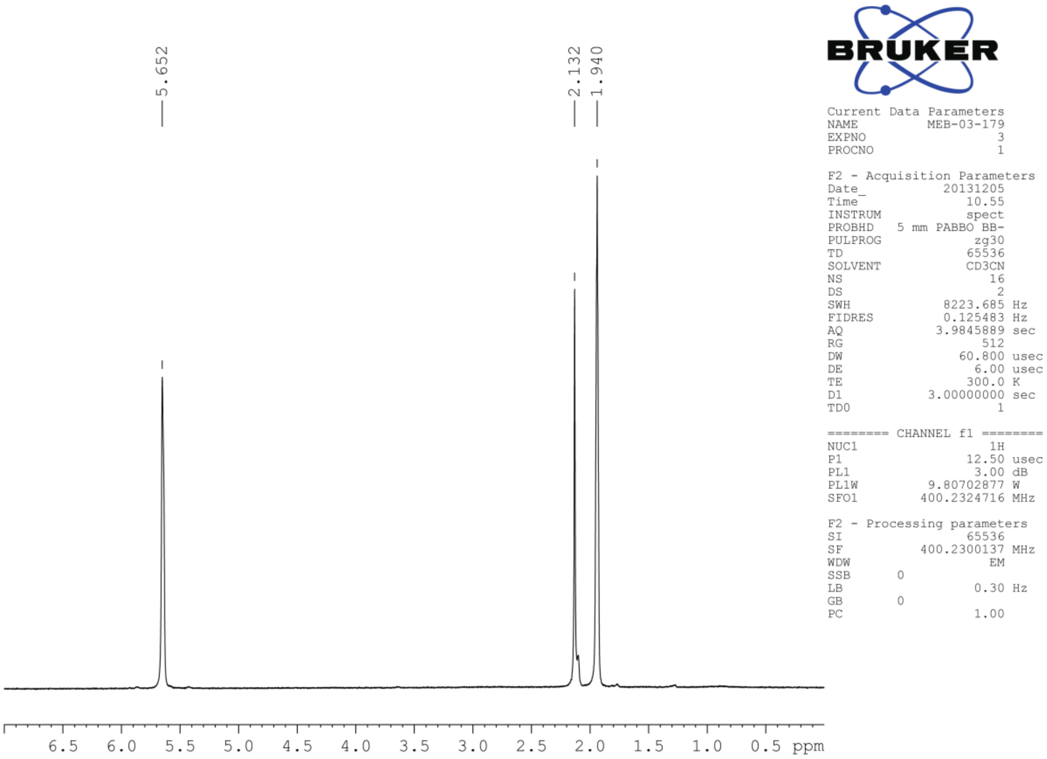

Figure ESI3.2.1: ^{1}H NMR of [^{99}Tc(benzene)$_2$](PF$_6$).

3.2.2 ^{13}C NMR [^{99}Tc(benzene)$_2$](PF$_6$)

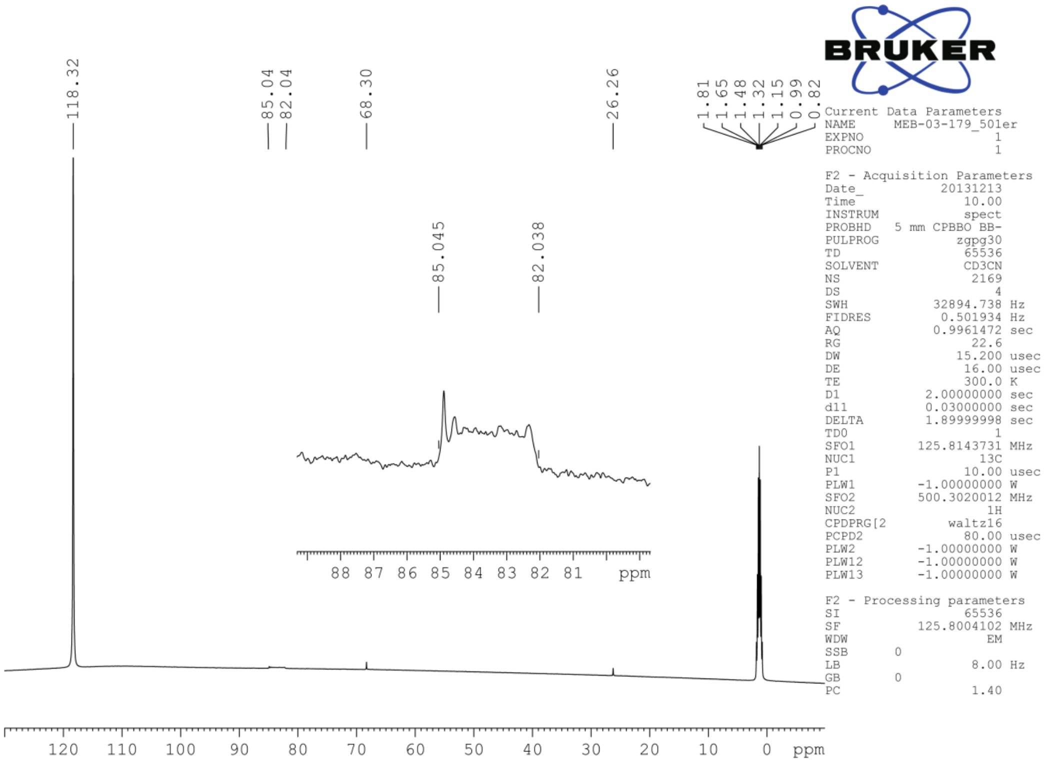

Figure ESI3.2.2: ^{13}C NMR of [^{99}Tc(benzene)$_2$](PF$_6$).

3.2.3 ^{99}Tc NMR [^{99}Tc(benzene)$_2$](PF$_6$)

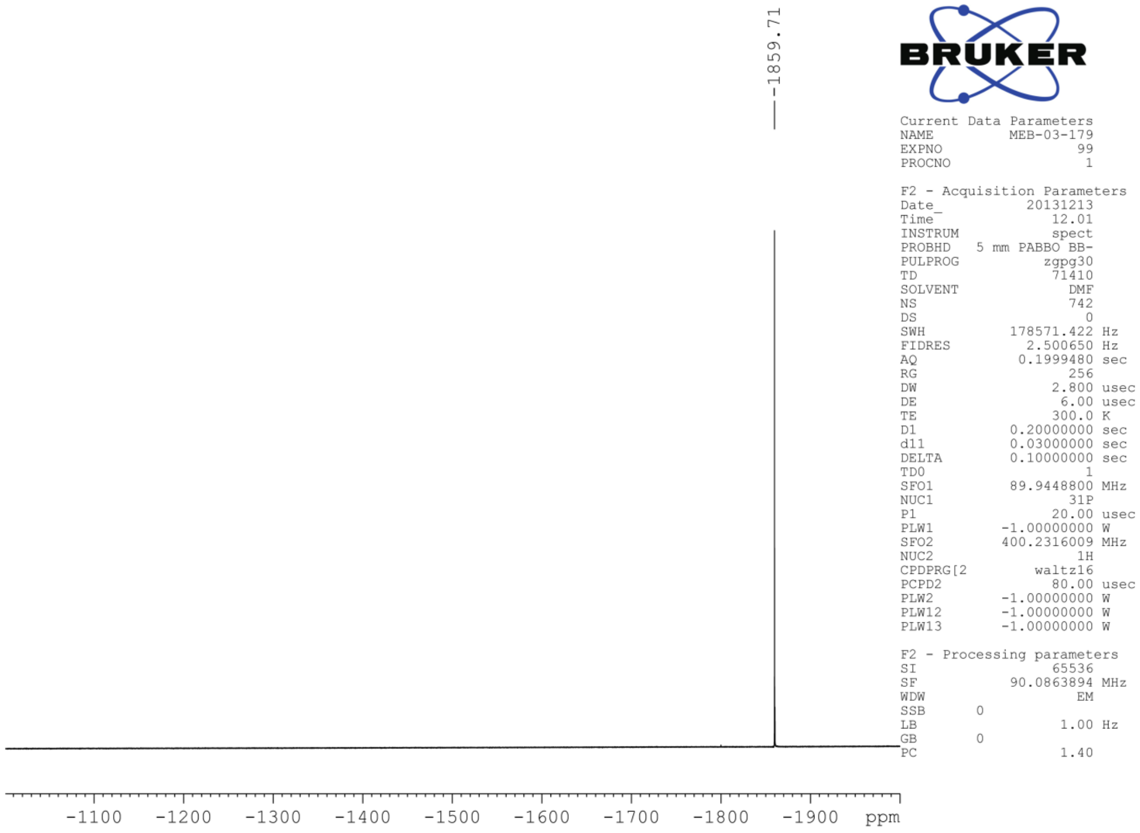

Figure ESI3.2.3: ^{99}Tc NMR of [^{99}Tc(benzene)$_2$](PF$_6$).

3.3 [^{99}Tc(toluene)$_2$](PF$_6$) ([2](PF$_6$))

3.3.1 ^{1}H NMR [^{99}Tc(toluene)$_2$](PF$_6$)

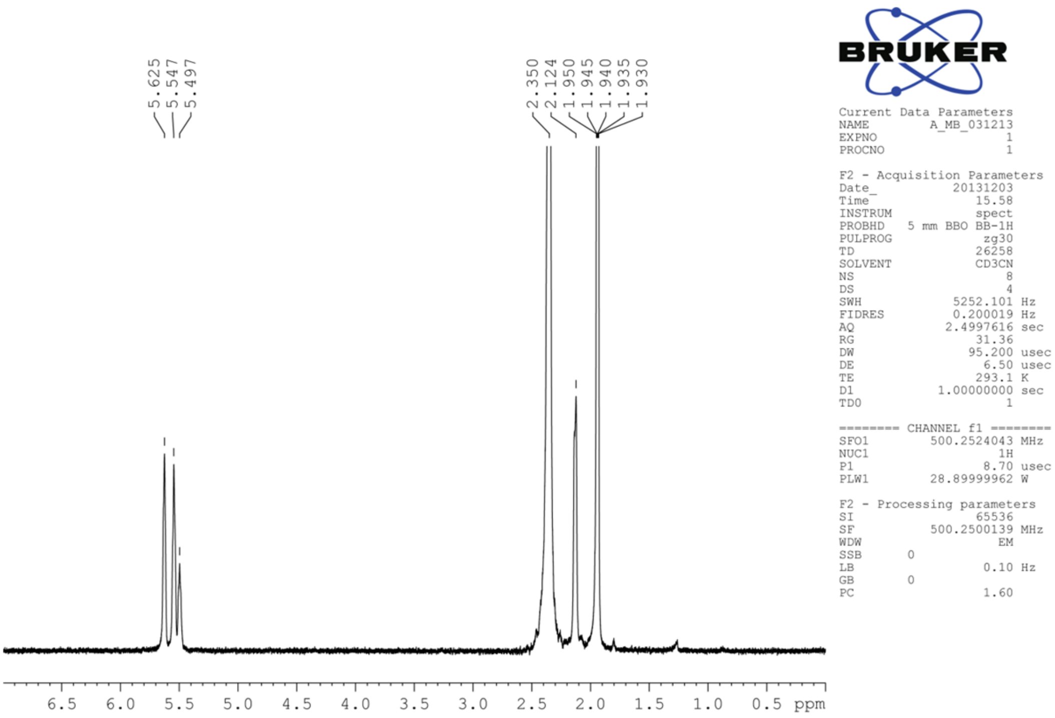

Figure ESI3.3.1: ^{1}H NMR of [^{99}Tc(toluene)$_2$](PF$_6$).

3.3.2 ^{13}C NMR [^{99}Tc(toluene)$_2$](PF$_6$)

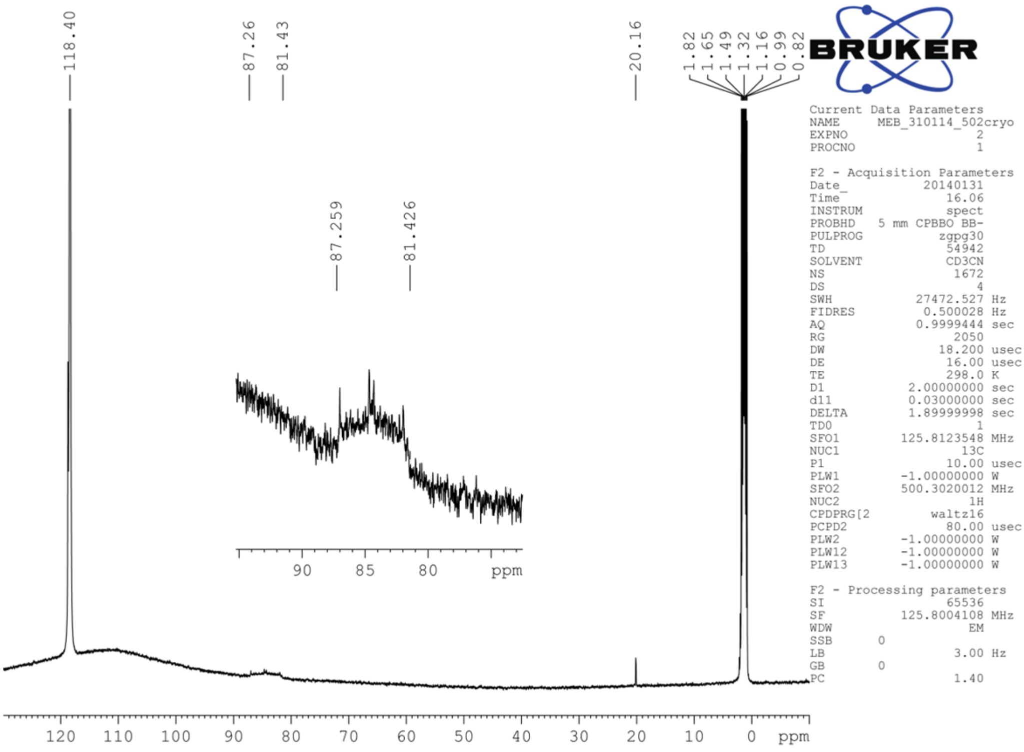

Figure ESI3.3.2: ^{13}C NMR of [^{99}Tc(toluene)$_2$](PF$_6$).

3.3.3 ^{99}Tc NMR [^{99}Tc(toluene)$_2$](PF$_6$)

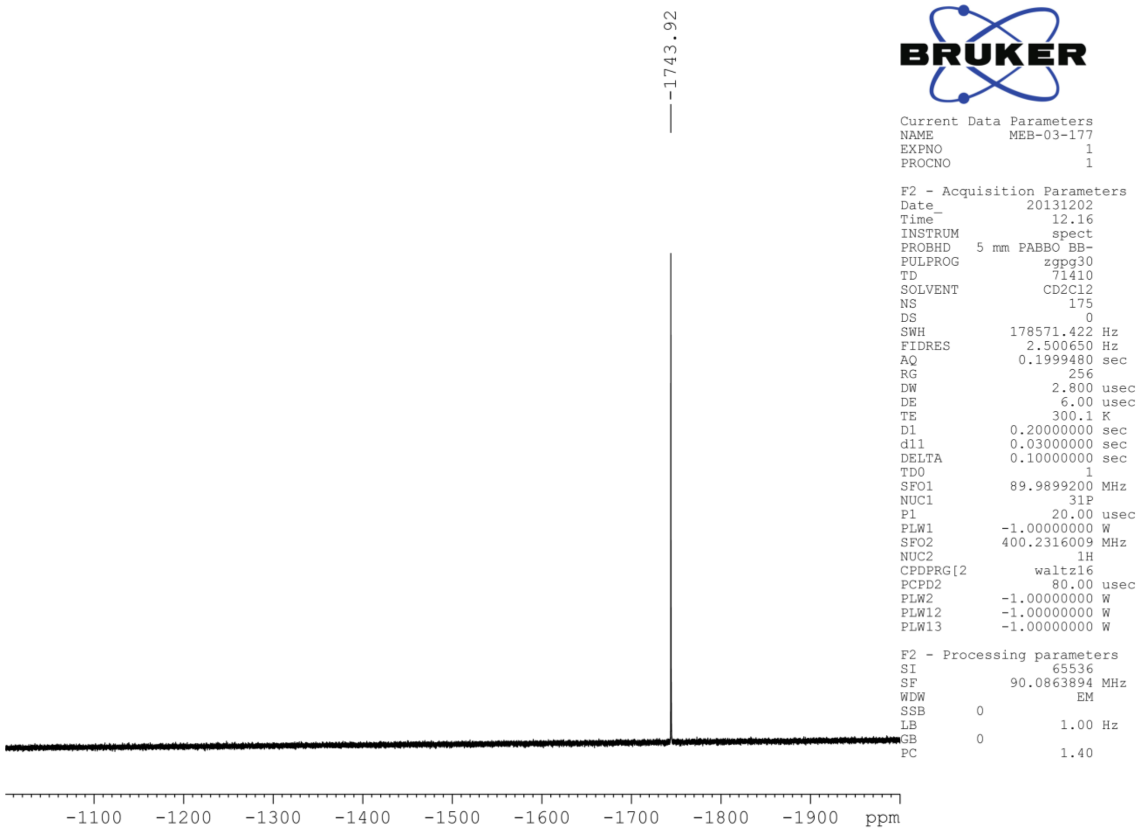

Figure ESI3.3.3: ^{99}Tc NMR of [^{99}Tc(toluene)$_2$](PF$_6$).

3.4 [^{99}Tc(mesitylene)$_2$](PF$_6$) ([3](PF$_6$))

3.4.1 ^{1}H NMR [^{99}Tc(mesitylene)$_2$](PF$_6$)

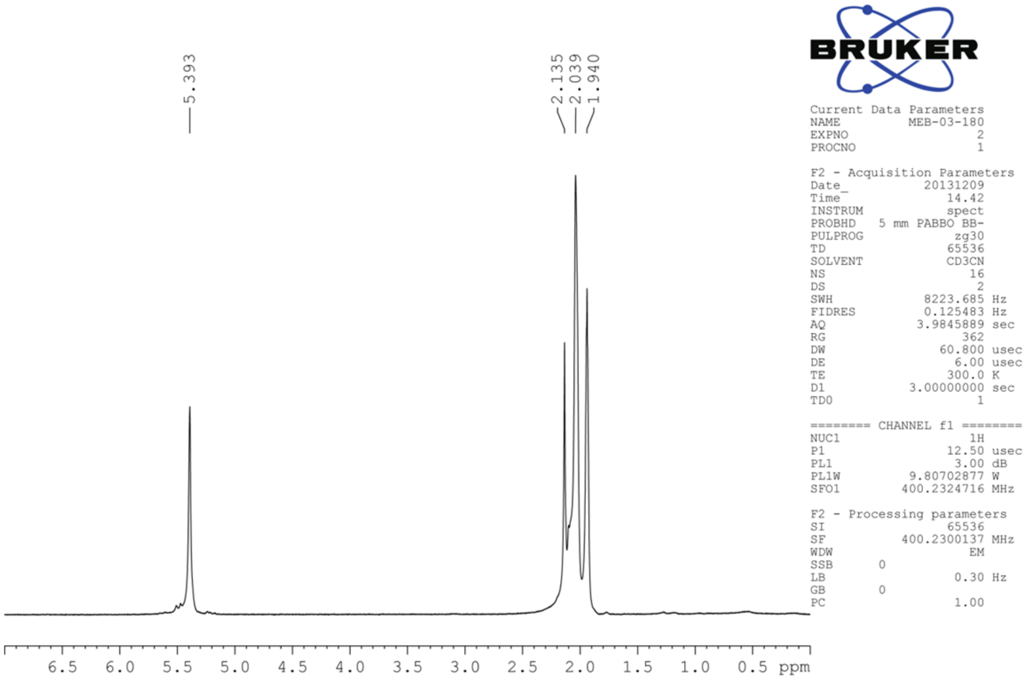

Figure ESI3.4.1: ^{1}H NMR of [^{99}Tc(mesitylene)$_2$](PF$_6$).

3.4.2 ^{13}C NMR [^{99}Tc(mesitylene)$_2$](PF$_6$)

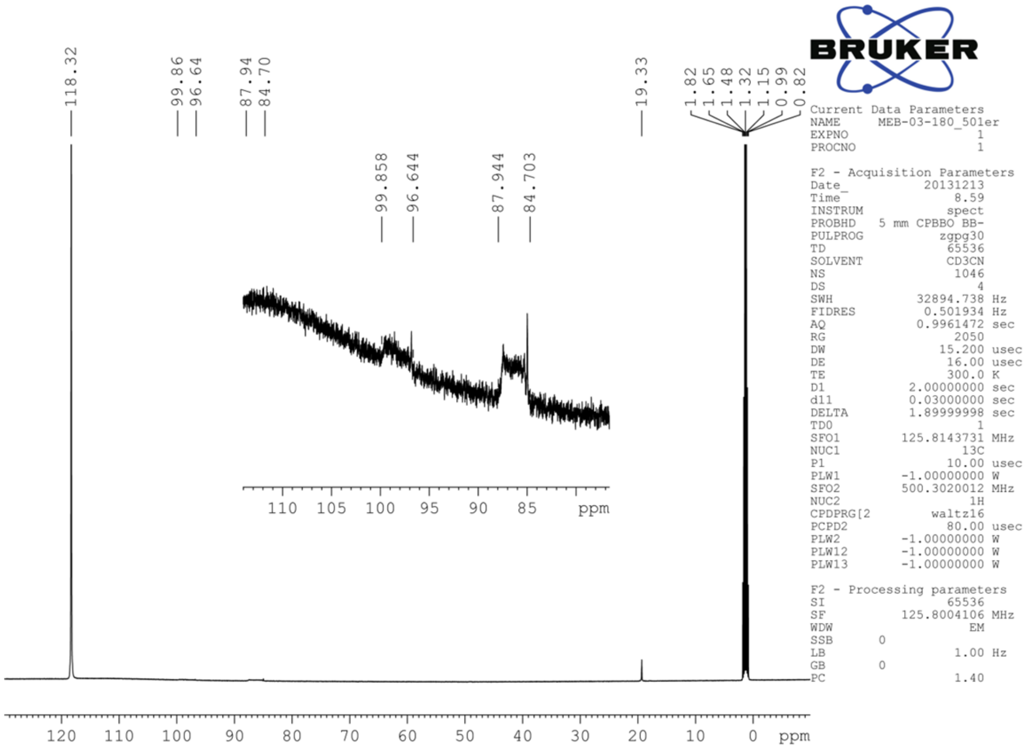

Figure ESI3.4.2: ^{13}C NMR of [^{99}Tc(mesitylene)$_2$](PF$_6$).

3.4.3 ^{99}Tc NMR [^{99}Tc(mesitylene)$_2$](PF$_6$)

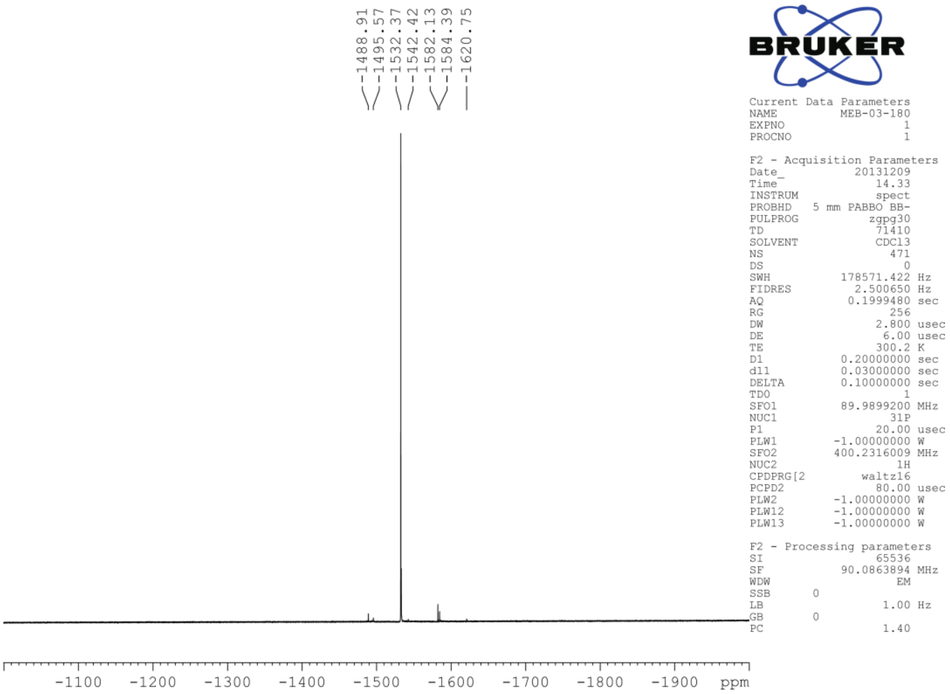

Figure ESI3.4.3: ^{99}Tc NMR of [^{99}Tc(mesitylene)$_2$](PF$_6$).

3.5 [^{99}Tc(tetralin)$_2$](PF$_6$) ([4](PF$_6$))

3.5.1 ^{1}H NMR [^{99}Tc(tetralin)$_2$](PF$_6$)

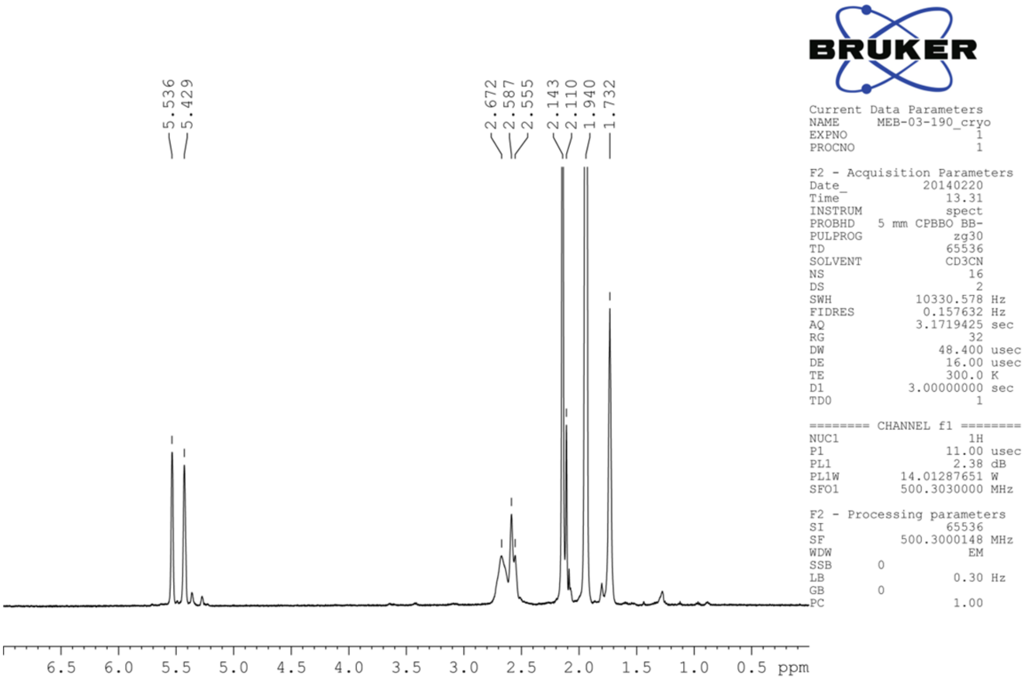

Figure ESI3.5.1: ^{1}H NMR of [^{99}Tc(tetralin)$_2$](PF$_6$).

3.5.2 ^{13}C NMR [^{99}Tc(tetralin)$_2$](PF$_6$)

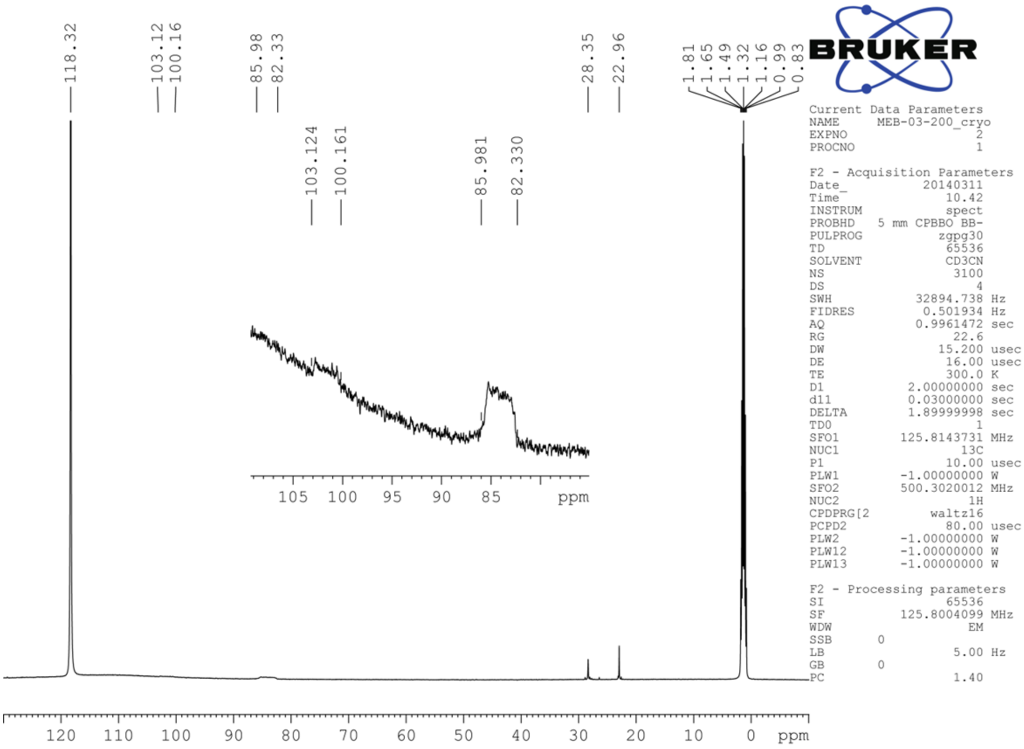

Figure ESI3.5.2: ^{13}C NMR of [^{99}Tc(tetralin)$_2$](PF$_6$).

3.5.3 ^{99}Tc NMR [^{99}Tc(tetralin)$_2$](PF_6)

^{99}Tc NMR of the product mixture [^{99}Tc(tetralin)$_2$](PF_6) + [^{99}Tc(tetralin)(OHPhen)]$^+$ (OHPhen = 1,2,3,4,5,6,7,8-octahydrophenanthrene)

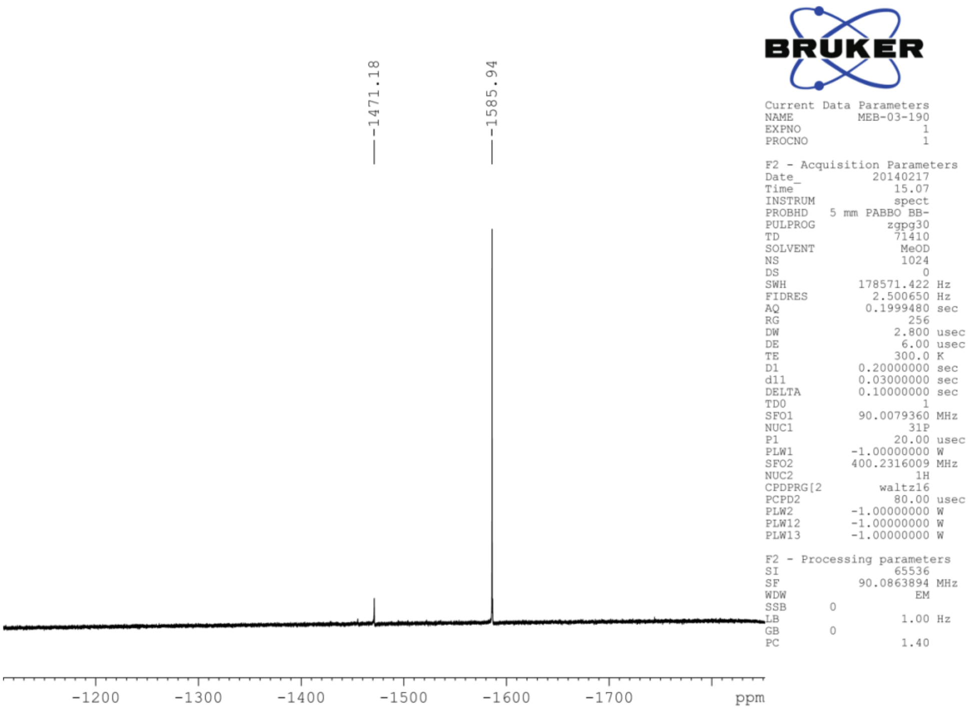

Figure ESI3.5.3.1: ^{99}Tc NMR of [^{99}Tc(tetralin)$_2$](PF_6) + [^{99}Tc(tetralin)(OHPhen)]$^+$.

^{99}Tc NMR of the pure main product [^{99}Tc(tetralin)$_2$](PF_6)

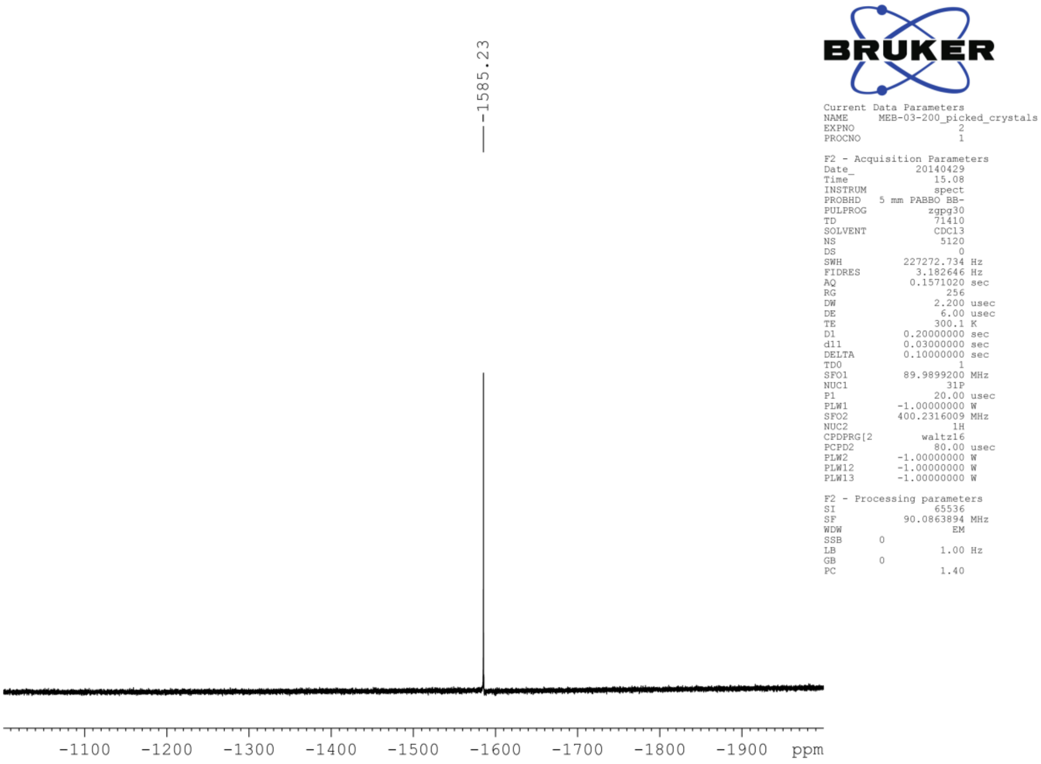

Figure ESI3.5.3.2: ^{99}Tc NMR pure main product [^{99}Tc(tetralin)$_2$](PF_6).

^{99}Tc NMR of the pure side product [^{99}Tc(tetralin)(OHPhen)]$^{+}$

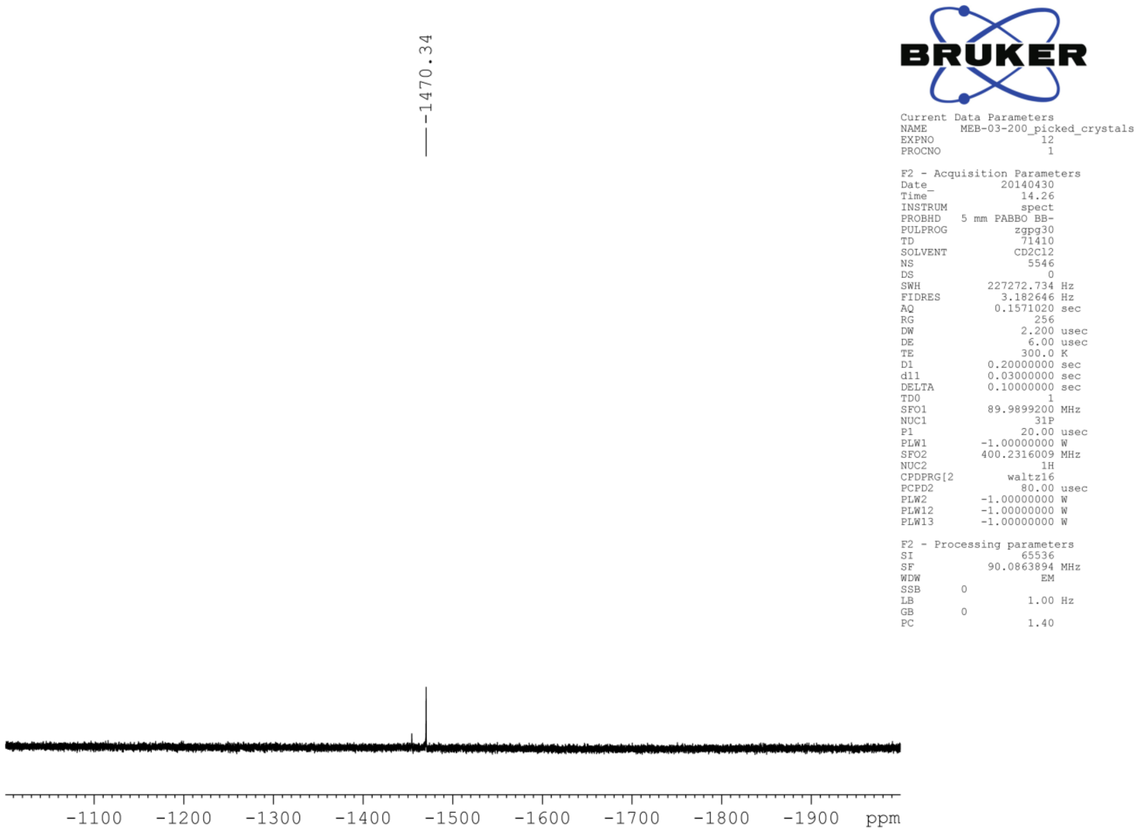

Figure ESI3.5.3.3: ^{99}Tc NMR pure side product [^{99}Tc(tetralin)(OHPhen)]$^{+}$.

4 Crystallographic Details

Crystallographic data were collected at 183(2) K with Mo Kα radiation (λ = 0.7107 Å) that was monochromated with help of graphite on an Oxford Diffraction Xcalibur system with a Ruby detector. Suitable crystals were covered with oil (Infineum V8512, formerly known as Paratone N), mounted on top of a loop and immediately transferred to the diffractometer. The program suite CrysAlis Pro was used for data collection, semi-empirical absorption correction, and data reduction.[11] More details on data collection and structure calculations are contained in table ESI4.1. Structures were solved with direct methods using SIR97[12] and were refined by full-matrix least-squares methods on F^2 with SHELXL-97. The refinements have been done with anisotropic thermal parameters for all non-hydrogen atoms. The positions of the hydrogen atoms have been calculated using the 'riding model' option of SHELXL97.[13]

Table ESI4.1: Data collection and structure calculation details.

	$[^{99}Tc(benzene)_2](PF_6)$ [**1**](PF_6)	$[^{99}Tc(toluene)_2](PF_6)$ [**2**](PF_6)	$[^{99}Tc(mesitylene)_2](PF_6)$ [**3**](PF_6)
Formula	$C_{12}H_{12}F_6PTc$	$C_{14}H_{16}F_6PTc$	$C_{18}H_{24}F_6PTc$
M_w / g mol^{-1}	400.19	428.24	484.34
Space group	$P2_1/c$	$P2_1/n$	$P2_12_12_1$
Temperature / K	183	183	183
a / Å	13.8831(7)	7.7559(8)	9.2697(5)
b / Å	9.8745(5)	11.0030(8)	13.0695(16)
c / Å	9.7190(6)	8.9353(8)	15.815(6)
α / °	90	90	90
β / °	94.232(5)	98.474(9)	90
γ / °	90	90	90
μ / mm^{-1}	1.260	1.116	0.89
Z	4	2	4
V / Å^3	1328.73(13)	754.20(12)	1916.0(8)
ρ_{calc} / g cm^{-3}	2.000	1.885	1.676
Crystal description	colorless block	colorless block	colorless block
Θ range [°]	2.5 to 28.3	2.9 to 29.3	2.8 to 32.6
Index ranges	-14<=h<=18, -13<=k<=12,	-7<=h<=9, -13<=k<=13,	-13<=h<=12, -18<=k<=18,

	-9<=l<=12	-11<=l<=11	-22<=l<=20
Refl collected	7418	5202	12954
Indep refl	3296	1545	5851
Refl obs I > 2σ(I)	2139 [R(int) = 0.026]	1125 [R(int) = 0.062]	5484 [R(int) = 0.032]
Compl to theta	99.9 % to 28.3°	100% to 26.4°	100% to 30.5°
Max. and min. transm	0.824 and 0.719	0.906 and 0.649	0.908 and 0.688
Restraints / param	0 / 184	0 / 104	0 / 277
Goodness-of-fit on F^2	1.02	1.04	1.03
Final diff ρ_{max} (e^-/ $Å^3$)	0.38 and -0.61	1.10 and -0.40	0.88 and -0.46
R1[a, c]	0.032	0.041	0.034
wR2[b, c]	0.09	0.098	0.064
CCDC	1017765	1017766	1017767

[a] $R1 = |F_o - F_c|/|F_o|$; [b] $wR2 = [w(F_o^2 – F_c^2)^2/(wF_o^2)]^{1/2}$; [c] I > 2 sig(I)

	$[^{99}Tc(tetralin)_2](PF_6)$ **[4]**(PF_6)	$[Al(dmf)_6][^{99}TcCl_6]Cl\cdot(dmf)$
Formula	$C_{20}H_{24}F_6PTc$	$C_{21}H_{49}AlCl_7N_7O_7Tc$
M_w / g mol^{-1}	508.36	885.71
Space group	$P2_1/n$	$P2_1$
Temperature / K	183	183
a / Å	8.7563(6)	8.8724(3)
b / Å	12.1899(11)	23.5963(7)
c / Å	9.5187(15)	9.6318(3)
α / °	90	90
β / °	108.089(10)	96.888(3)
γ / °	90	90
μ / mm^{-1}	0.89	0.89
Z	2	2
V / Å^3	965.8(2)	2001.92(11)
ρ_{calc} / g cm^{-3}	1.745	1.468
Crystal description	yellow block	yellow block
Θ range [°]	3.2 to 32.5	2.9 to 31.8
Index ranges	-9<=h<=12, -16<=k<=17, -13<=l<=12	-11<=h<=9, -29<=k<=29, -12<=l<=12
Refl collected	6611	18867
Indep refl	2906	8169
Refl obs I > 2σ(I)	2360 [R(int) = 0.022]	7662 [R(int) = 0.04]
Compl to theta	98.2 % to 30.5°	100 % to 26.4°
Max. and min. transm	0.850 and 0.632	0.879 and 0.515
Restraints / param	0 / 148	1 / 411
Goodness-of-fit on F^2	1.08	1.02
Final diff ρ_{max} (e^-/ Å^3)	1.53 and -0.46	0.60 and -0.37
R1[a, c]	0.038	0.031

wR2[b, c]	0.107	0.067
CCDC	1017768	1017769

[a] $R1 = |F_o - F_c|/|F_o|$; [b] $wR2 = [w(F_o^2 - F_c^2)^2/(wF_o^2)]^{1/2}$; [c] $I > 2\,sig(I)$

4.1 [^{99}Tc(benzene)$_2$](PF$_6$) ([1](PF$_6$))

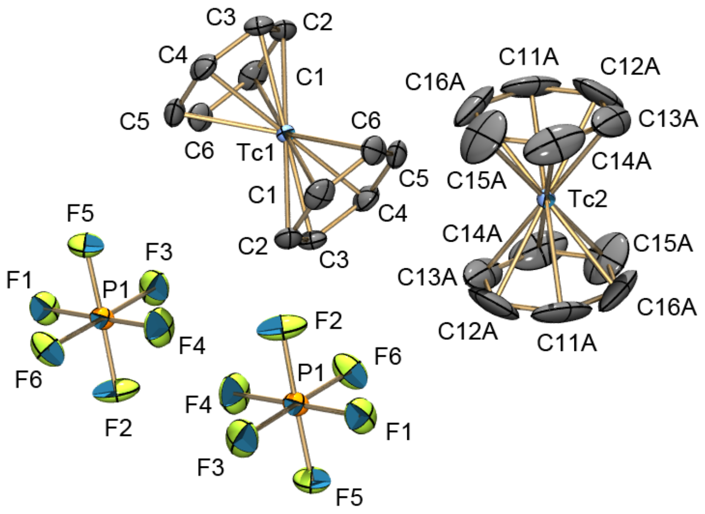

Bond lengths [Å] and angles [°] for [^{99}Tc(benzene)$_2$](PF$_6$)

Tc(1)-C(1)	2.217(3)	C(1)-Tc(1)-C(2)	36.23(15)
Tc(1)-C(2)	2.225(3)	C(2)-Tc(1)-C(3)	37.04(14)
Tc(1)-C(3)	2.215(3)	C(3)-Tc(1)-C(4)	37.04(13)
Tc(1)-C(4)	2.217(3)	C(4)-Tc(1)-C(5)	36.59(14)
Tc(1)-C(5)	2.216(3)	C(5)-Tc(1)-C(6)	36.73(13)
Tc(1)-C(6)	2.210(3)	C(6)-Tc(1)-C(1)	36.95(14)
Tc(2)-C(11A)	2.178(5)	C(11A)-Tc(2)-C(12A)	36.1(3)
Tc(2)-C(12A)	2.176(5)	C(12A)-Tc(2)-C(13A)	36.3(3)
Tc(2)-C(13A)	2.194(4)	C(13A)-Tc(2)-C(14A)	36.4(3)
Tc(2)-C(14A)	2.196(5)	C(14A)-Tc(2)-C(15A)	36.9(2)
Tc(2)-C(15A)	2.180(5)	C(15A)-Tc(2)-C(16A)	37.3(2)
Tc(2)-C(16A)	2.184(4)	C(16A)-Tc(2)-C(11A)	36.0(3)

4.2 [^{99}Tc(toluene)$_2$](PF$_6$) ([2](PF$_6$))

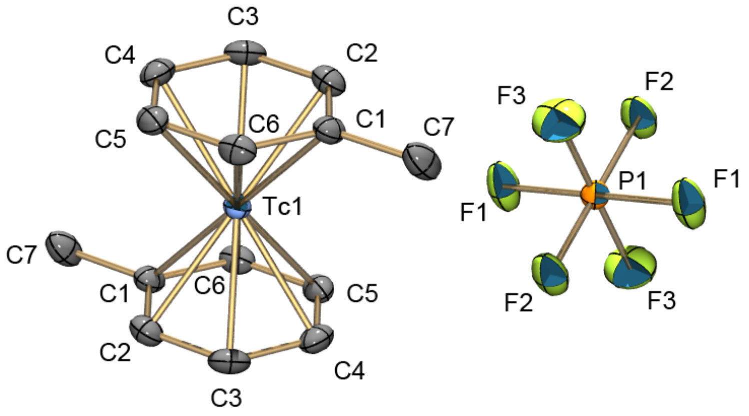

Bond lengths [Å] and angles [°] for [^{99}Tc(toluene)$_2$](PF$_6$)

Tc(1)-C(1)	2.245(4)	C(1)-Tc(1)-C(2)	36.83(14)
Tc(1)-C(2)	2.221(4)	C(2)-Tc(1)-C(3)	36.76(15)
Tc(1)-C(3)	2.225(4)	C(3)-Tc(1)-C(4)	36.76(14)
Tc(1)-C(4)	2.219(4)	C(4)-Tc(1)-C(5)	36.59(15)
Tc(1)-C(5)	2.217(4)	C(5)-Tc(1)-C(6)	36.83(14)
Tc(1)-C(6)	2.223(4)	C(6)-Tc(1)-C(1)	37.18(15)

4.3 [^{99}Tc(mesitylene)$_2$](PF$_6$) ([3](PF$_6$))

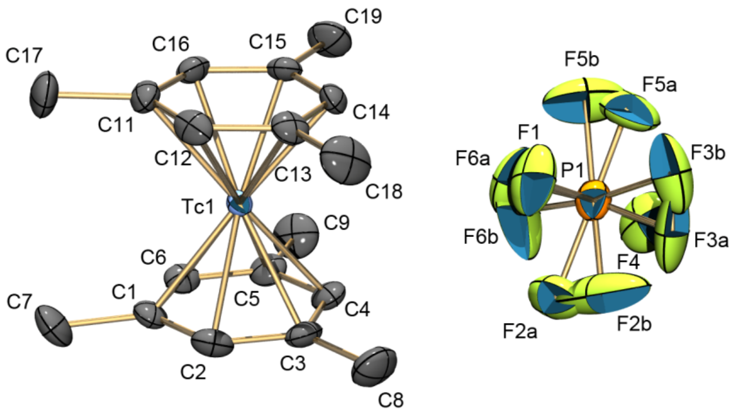

Bond lengths [Å] and angles [°] for [^{99}Tc(mesitylene)$_2$](PF$_6$)

Tc(1)-C(1)	2.245(3)	C(1)-Tc(1)-C(2)	37.14(10)
Tc(1)-C(2)	2.213(3)	C(2)-Tc(1)-C(3)	36.82(11)
Tc(1)-C(3)	2.243(3)	C(3)-Tc(1)-C(4)	36.83(10)
Tc(1)-C(4)	2.223(2)	C(4)-Tc(1)-C(5)	36.90(11)
Tc(1)-C(5)	2.248(3)	C(5)-Tc(1)-C(6)	36.49(10)
Tc(1)-C(6)	2.236(3)	C(6)-Tc(1)-C(1)	36.45(10)
Tc(1)-C(11)	2.250(2)	C(11)-Tc(1)-C(12)	37.01(11)
Tc(1)-C(12)	2.226(3)	C(12)-Tc(1)-C(13)	36.83(10)
Tc(1)-C(13)	2.237(3)	C(13)-Tc(1)-C(14)	37.05(11)
Tc(1)-C(14)	2.214(2)	C(14)-Tc(1)-C(15)	36.86(10)
Tc(1)-C(15)	2.241(3)	C(15)-Tc(1)-C(16)	36.88(10)
Tc(1)-C(16)	2.228(3)		

4.4 [^{99}Tc(tetralin)$_2$](PF$_6$) ([4](PF$_6$))

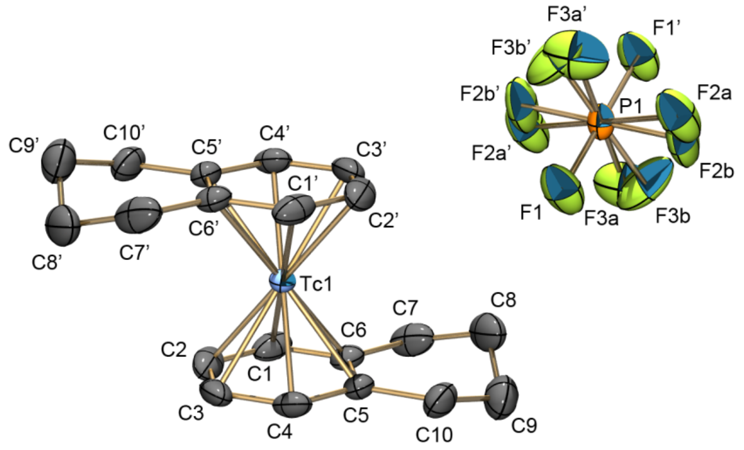

Bond lengths [Å] and angles [°] for [^{99}Tc(tetralin)$_2$](PF$_6$)

Tc(1)-C(1)	2.219(2)	C(1)-Tc(1)-C(2)	36.54(11)
Tc(1)-C(2)	2.228(2)	C(2)-Tc(1)-C(3)	36.29(12)
Tc(1)-C(3)	2.231(2)	C(3)-Tc(1)-C(4)	36.90(10)
Tc(1)-C(4)	2.227(2)	C(4)-Tc(1)-C(5)	37.03(9)
Tc(1)-C(5)	2.250(2)	C(5)-Tc(1)-C(6)	36.50(9)
Tc(1)-C(6)	2.250(2)	C(6)-Tc(1)-C(1)	37.31(9)

4.5 [^{99}Tc(tetralin)(OHPhen)]$^+$ (OHPhen = 1,2,3,4,5,6,7,8-octahydrophenanthrene, [5]$^+$)

Due to the weak diffraction of the [^{99}Tc(tetralin)(OHPhen)](PF_6) crystals, only data sets of low quality have been recorded. Consequently, the structure analysis of [^{99}Tc(tetralin)(OHPhen)](PF_6) had to be stopped after the structure solution (no structure refinement possible). The presentation shows the result of the structure solution.

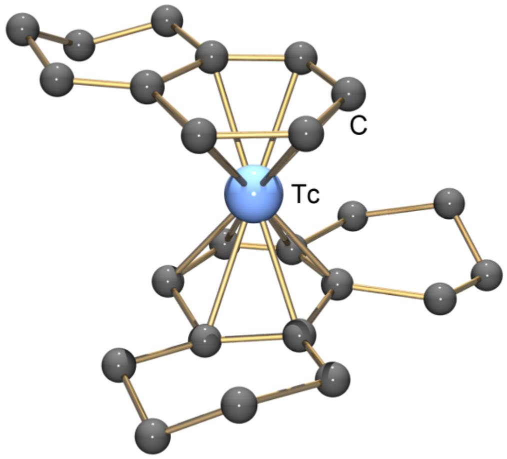

4.6 [Al(dmf)$_6$][^{99}TcCl$_6$]Cl·(dmf)

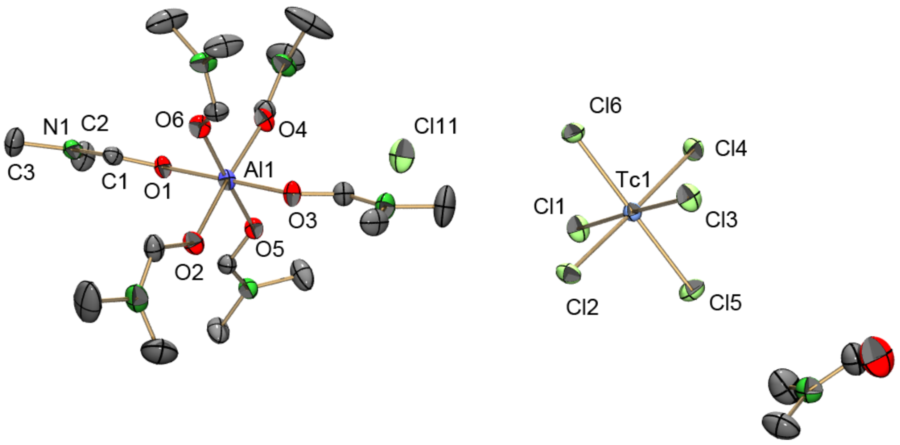

Bond lengths [Å] and angles [°] for [Al(dmf)$_6$][^{99}TcCl$_6$]Cl•(dmf)

Tc(1)-Cl(1)	2.3605(8)	Cl(1)-Tc(1)-Cl(2)	89.28(3)
Tc(1)-Cl(2)	2.3580(8)	Cl(1)-Tc(1)-Cl(3)	178.18(3)
Tc(1)-Cl(3)	2.3531(7)	Cl(1)-Tc(1)-Cl(4)	91.08(3)
Tc(1)-Cl(4)	2.3549(8)	Cl(1)-Tc(1)-Cl(5)	88.68(3)
Tc(1)-Cl(5)	2.3577(7)	Cl(1)-Tc(1)-Cl(6)	91.80(3)
Tc(1)-Cl(6)	2.3523(7)	Cl(2)-Tc(1)-Cl(3)	89.65(3)
Al(1)-O(1)	1.872(2)	Cl(2)-Tc(1)-Cl(4)	178.23(3)
Al(1)-O(2)	1.890(2)	Cl(2)-Tc(1)-Cl(5)	91.68(3)
Al(1)-O(3)	1.881(2)	Cl(2)-Tc(1)-Cl(6)	88.48(3)
Al(1)-O(4)	1.892(2)	Cl(3)-Tc(1)-Cl(4)	90.04(3)
Al(1)-O(5)	1.875(2)	Cl(3)-Tc(1)-Cl(5)	89.88(3)
Al(1)-O(6)	1.862(2)	Cl(3)-Tc(1)-Cl(6)	89.64(3)
		Cl(4)-Tc(1)-Cl(5)	90.06(3)
		Cl(4)-Tc(1)-Cl(6)	89.78(3)
		Cl(5)-Tc(1)-Cl(6)	179.49(3)
		O(1)-Al(1)-O(2)	92.83(9)
		O(1)-Al(1)-O(3)	179.22(11)
		O(1)-Al(1)-O(4)	90.18(10)
		O(1)-Al(1)-O(5)	89.36(9)
		O(1)-Al(1)-O(6)	88.90(9)
		O(2)-Al(1)-O(3)	86.44(10)
		O(2)-Al(1)-O(4)	176.39(11)
		O(2)-Al(1)-O(5)	89.13(10)
		O(2)-Al(1)-O(6)	94.26(10)
		O(3)-Al(1)-O(4)	90.56(10)
		O(3)-Al(1)-O(5)	90.90(10)
		O(3)-Al(1)-O(6)	90.89(9)
		O(4)-Al(1)-O(5)	88.92(10)
		O(4)-Al(1)-O(6)	87.79(10)
		O(5)-Al(1)-O(6)	176.26(11)

5 CV Data

5.1 Overview CV Data

Table ESI5.1.1: Overview CV data (Against Fc/Fc^+ at 0.45 V in MeCN unless stated otherwise)

Compound	Reduction Potential [V]	Oxidation Potential [V]
$[^{99}Tc(benzene)_2](PF_6)$[1]	-2.04	1.53
$[^{99}Tc(toluene)_2](PF_6)$[1]	-2.12	1.47
$[^{99}Tc(mesitylene)_2](PF_6)$[1]	-2.27	1.38
$[^{99}Tc(tetralin)_2](PF_6)$[1]	-2.15	1.39
$[Re(benzene)_2](PF_6)$[1]	-2.00	1.33
$[Re(toluene)_2](OTf)$[1]	-2.07	1.30
$[Re(mesitylene)_2](PF_6)$[1]	-2.24	1.23
$[Re(tetralin)_2](OTf)$[1]	-2.13	1.24
$[Fe(C_6Me_6)_2](PF_6)_2$[2]	-0.62, -1.82	-
$[Ru(benzene)_2](BF_4)_2$[a, 3]	-1.02	-
$[Ru(C_6Me_6)_2](BF_4)_2$[2]	-1.36	-
$[Os(C_6Me_6)_2](BF_4)_2$[2]	-1.25	-
$[Cr(benzene)_2]$[4]	-	0.82
$[Cr(benzene)_2]$[b, 4]	-	0.97
$[Cr(toluene)_2]$[b, 4]	-	1.04
$[Cr(mesitylene)_2]$[b, 4]	-	1.06

[a] $Ag/AgNO_3$ (0.1 M $AgNO_3$), 0.1 M $N^nBu_4ClO_4$ in MeCN, glassy carbon; [b] in H_2O, reference and conditions unclear.

Table ESI5.1.2: Overview CV-data

Compound	Reduction potential [V]	Oxidation potential [V]
$[^{99}Tc(benzene)_2](PF_6)$ ([**1**](PF_6))	-2.0360[a]	1.5215[b]
$[^{99}Tc(toluene)_2](PF_6)$ ([**2**](PF_6))	-2.1201[a]	1.4702[b]
$[^{99}Tc(mesitylene)_2](PF_6)$ ([**3**](PF_6))	-2.2706[a]	1.3799[b]
$[^{99}Tc(tetralin)_2](PF_6)$ ([**4**](PF_6))	-2.1538[a]	1.3876[b]

[a] irreversible reduction; [b] reversible oxidation.

5.2 [^{99}Tc(benzene)$_2$](PF$_6$) ([1](PF$_6$))

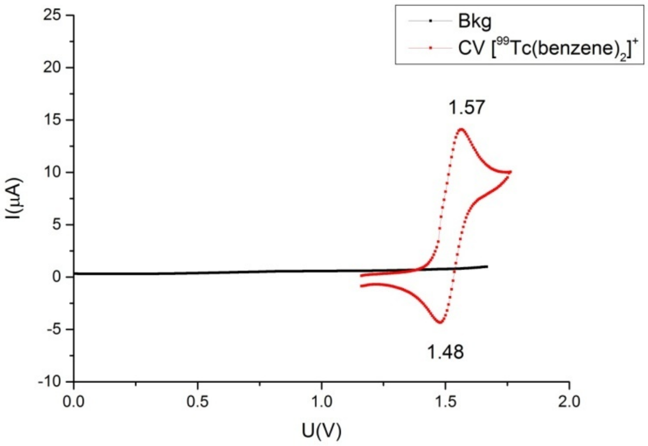

Figure ESI5.2.1: Oxidation of [^{99}Tc(benzene)$_2$](PF$_6$).

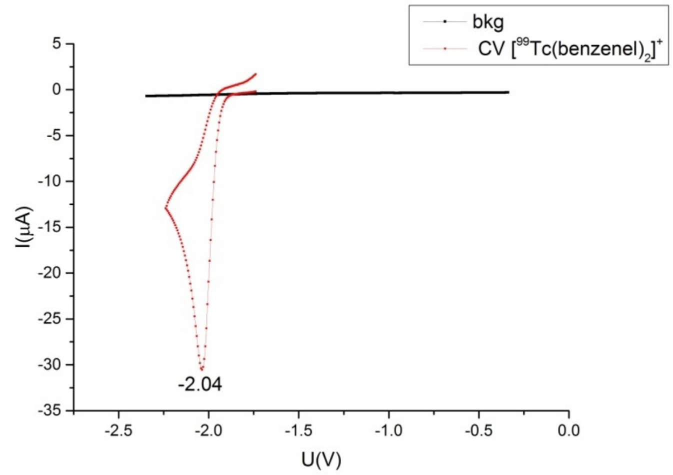

Figure ESI5.2.2: Reduction of [^{99}Tc(benzene)$_2$](PF$_6$).

5.3 [^{99}Tc(toluene)$_2$](PF$_6$) ([2](PF$_6$))

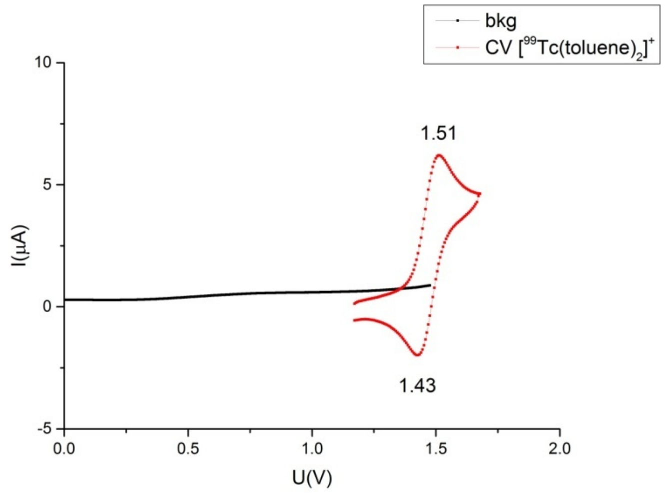

Figure ESI5.3.1: Oxidation of [^{99}Tc(toluene)$_2$](PF$_6$).

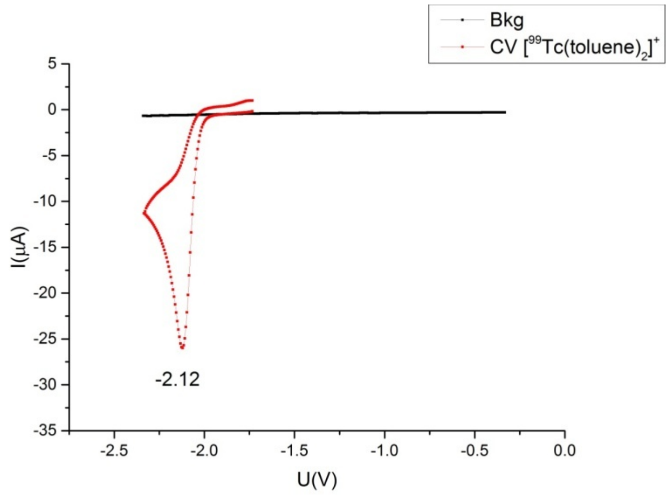

Figure ESI5.3.2: Reduction of [^{99}Tc(toluene)$_2$](PF$_6$).

5.4 [^{99}Tc(mesitylene)$_2$](PF$_6$) ([3](PF$_6$))

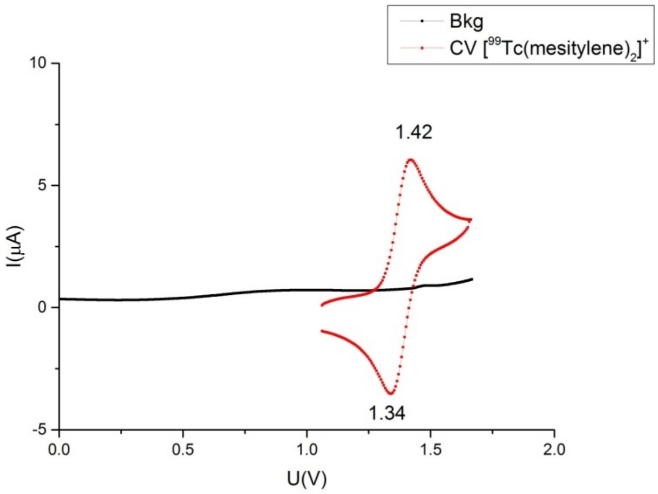

Figure ESI5.4.1: Oxidation of [^{99}Tc(mesitylene)$_2$](PF$_6$).

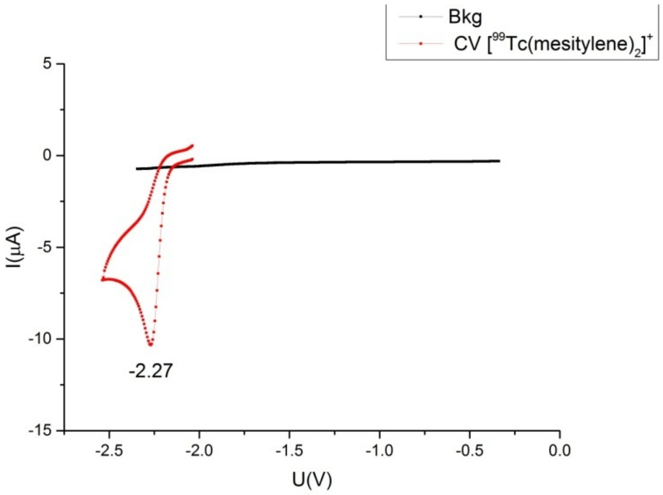

Figure ESI5.4.2: Reduction of [^{99}Tc(mesitylene)$_2$](PF$_6$).

5.5 [^{99}Tc(tetralin)$_2$](PF$_6$) ([4](PF$_6$))

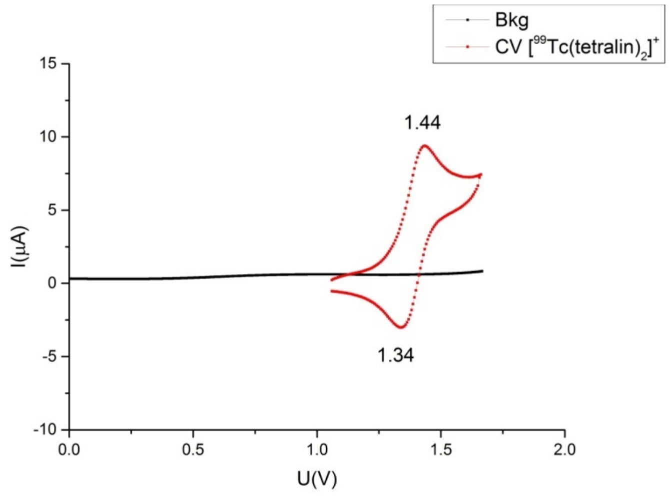

Figure ESI5.5.1: Oxidation of [^{99}Tc(tetralin)$_2$](PF$_6$).

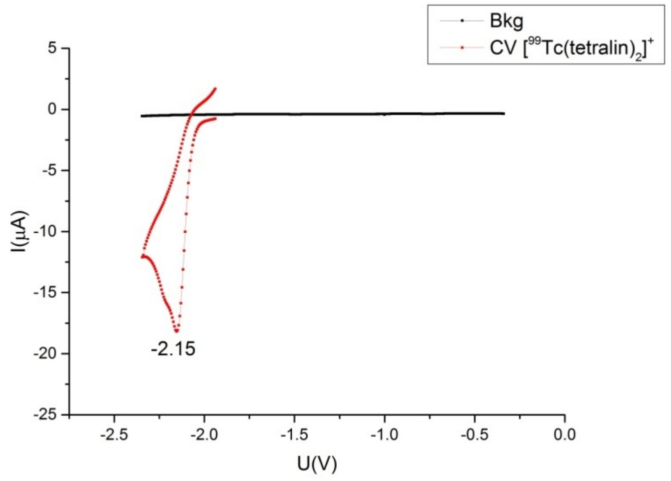

Figure ESI5.5.2: Reduction of [^{99}Tc(tetralin)$_2$](PF$_6$).

5.6 [Re(benzene)$_2$](PF$_6$)

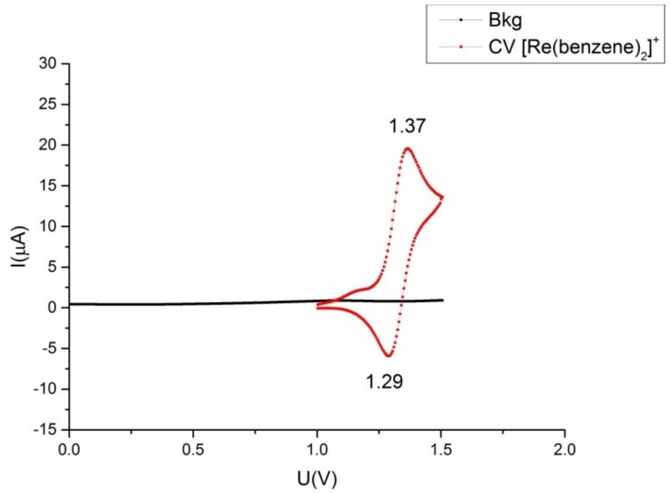

Figure ESI5.6.1: Oxidation of [Re(benzene)$_2$](PF$_6$).

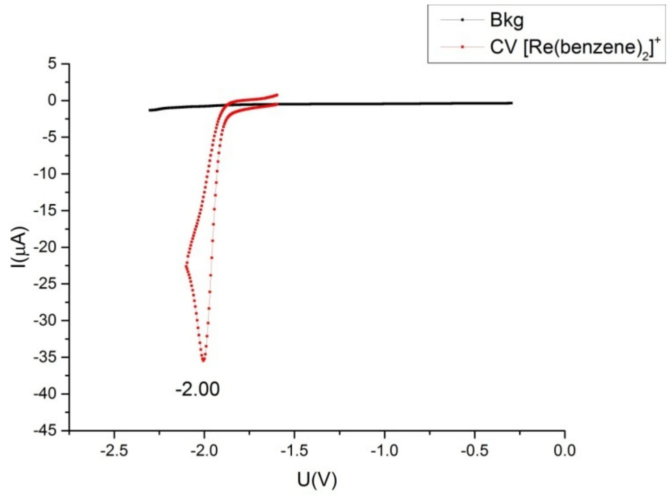

Figure ESI5.6.2: Reduction of [Re(benzene)$_2$](PF$_6$).

5.7 [Re(toluene)$_2$](OTf)

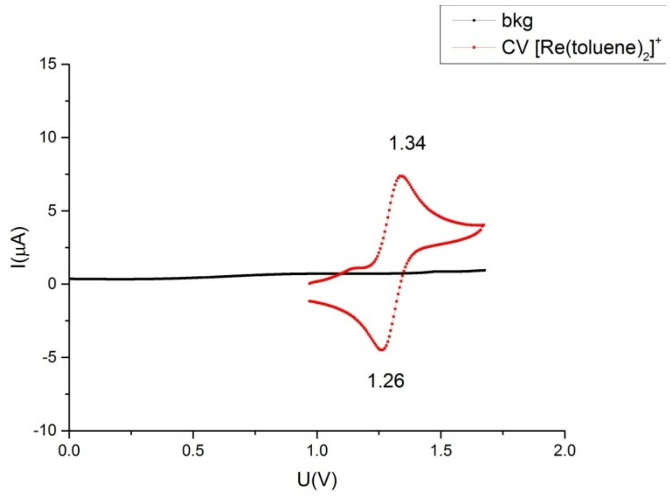

Figure ESI5.7.1: Oxidation of [Re(toluene)$_2$](OTf)

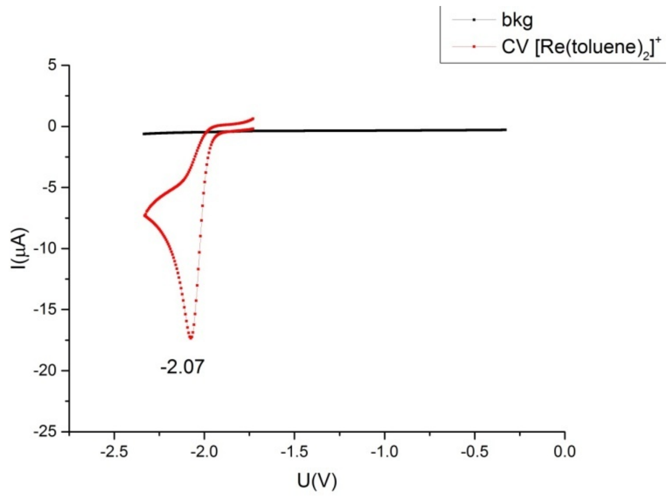

Figure ESI5.7.2: Reduction of [Re(toluene)$_2$](OTf)

5.8 [Re(mesitylene)$_2$](PF$_6$)

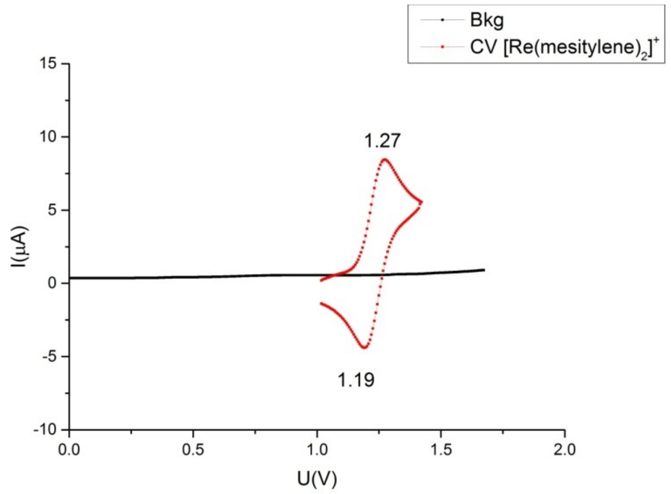

Figure ESI5.8.1: Oxidation of [Re(mesitylene)$_2$](PF$_6$)

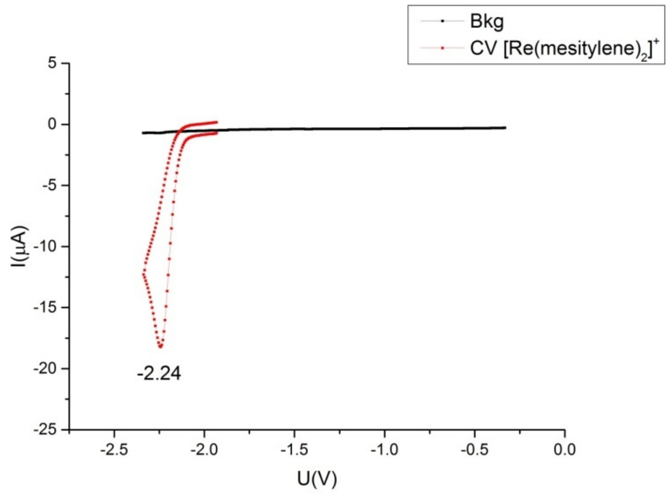

Figure ESI5.8.2: Reduction of [Re(mesitylene)$_2$](PF$_6$)

5.9 [Re(tetralin)$_2$](OTf)

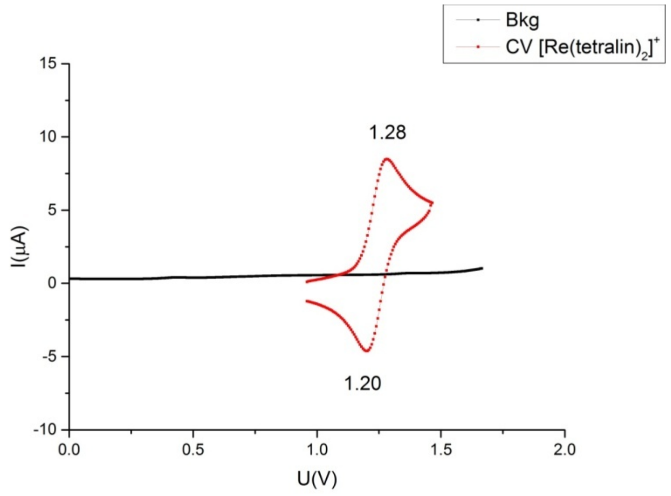

Figure ESI5.9.1: Oxidation of [Re(tetralin)$_2$](OTf)

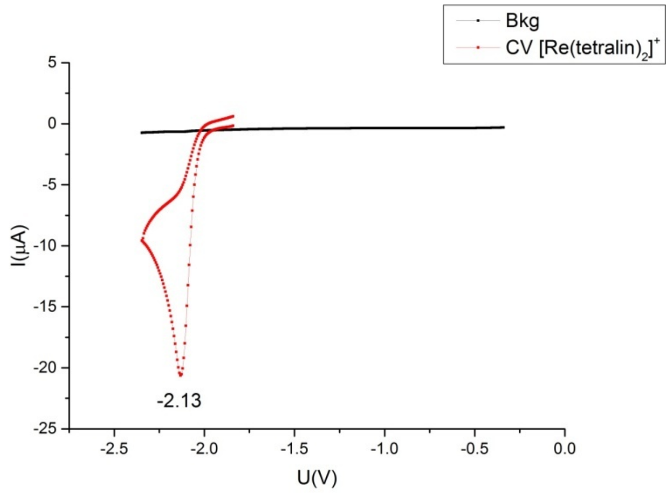

Figure ESI5.9.2: Reduction of [Re(tetralin)$_2$](OTf)

5.10 Spectroelectrochemistry of $[Re(toluene)_2]^+$ (OTTLE cell)

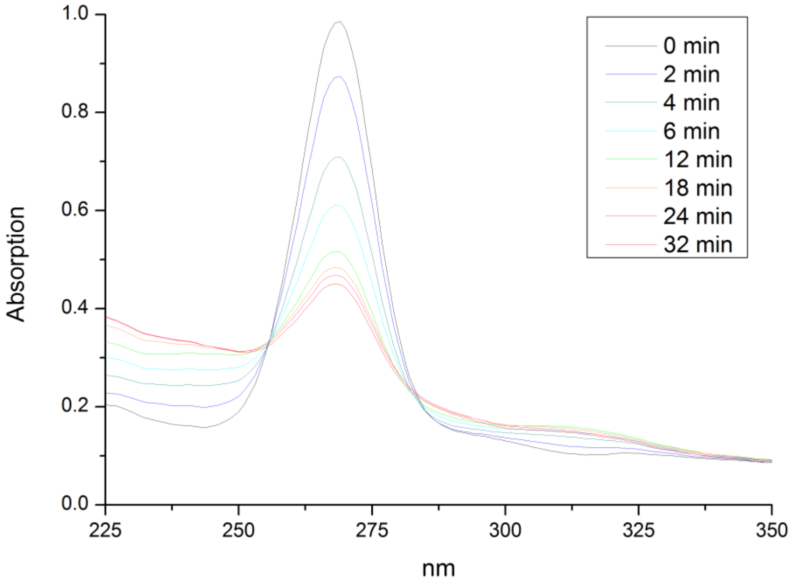

Figure ESI5.10.1: Spectroelectrochemical oxidation of $[Re(toluene)_2]^+$. Applied potential: 1.4 V; reference electrode: Ag/AgCl.

References

[1] This work

[2] Lord, R. L.; Schauer, C. K.; Schultz, F. A.; Baik, M.-H. *J. Am. Chem. Soc.* **2011**, *133*, 18234-18242.

[3] Satou, T.; Takehara, K.; Hirakida, M.; Sakamoto, Y.; Takemura, H.; Miura, H.; Tomonou, M.; Shinmyozu, T. *J. Organomet. Chem.* **1999**, *577*, 58-68.

[4] Yur'eva, L. P.; Nekrasov, L. N.; Peregudova, S. M. *Russ. Chem. Rev.* **1993**, *62*, 121.

[5] Trifonova, E. A.; Perekalin, D. S.; Lyssenko, K. A.; Kudinov, A. R. *J. Organomet. Chem.* **2013**, *727*, 60-63.

[6] Stibr, B.; Bakardjiev, M.; Hajkova, Z.; Holub, J.; Padelkova, Z.; Ruzicka, A.; Kennedy, J. D. *Dalton Trans.* **2011**, *40*, 5916-5920.

[7] Fischer, E. O.; Elschenbroich, C. *Chem. Ber.* **1970**, *103*, 162-172.

[8] Bennett, M. A.; Bown, M.; Byrnes, M. J. *J. Organomet. Chem.* **1998**, *571*, 139-144.

[9] Phillips, L.; Dennis, G. R.; Aroney, M. J. *New J. Chem.* **2000**, *24*, 27-32.

[10] Silvon, M. P.; Van Dam, E. M.; Skell, P. S. *J. Am. Chem. Soc.* **1974**, *96*, 1945-1946.

[11] *CrysAlis^{Pro} Software system*, vers. 171.32 Oxford; Oxford Diffraction Ltd.: UK, **2007**.

[12] Altomare, A.; Burla, M. C.; Camalli, M.; Cascarano, G. L.; Giacovazzo, C.; Guagliardi, A.; Moliterni, A. G. G.; Polidori, G.; Spagna, R., *J. Appl. Cryst.* **1999**, 32, 115-119.

[13] Sheldrick, G., *Acta Crystallogr. A* **2008**, 64, 112-122.
